# Genetic Variability of Chikungunya Virus in Southern Mexico

**DOI:** 10.3390/v11080714

**Published:** 2019-08-05

**Authors:** Kame A. Galan-Huerta, Viviana C. Zomosa-Signoret, Román Vidaltamayo, Sandra Caballero-Sosa, Ildefonso Fernández-Salas, Javier Ramos-Jiménez, Ana M. Rivas-Estilla

**Affiliations:** 1 Departamento de Bioquímica y Medicina Molecular, Facultad de Medicina, Universidad Autónoma de Nuevo Leon, Av. Francisco I. Madero S/N, Mitras Centro, Monterrey, Nuevo Leon 64460, Mexico; 2 Departamento de Ciencias Básicas. Escuela de Medicina, Universidad de Monterrey, Av. Morones Prieto No. 4500 pte, San Pedro Garza García, Nuevo Leon 64238, Mexico; 3 Clínica Hospital Dr. Roberto Nettel Flores, Instituto de Seguridad y Servicios Sociales de los Trabajadores del Estado, Av. Tuxtepec y Oaxaca S/N, Francisco Villa, Tapachula, Chiapas 30740, Mexico; 4 Centro Regional de Investigación en Salud Publica, Instituto Nacional de Salud Publica 4a Avenida Norte, esquina con calle 19 poniente S/N, Centro, Tapachula, Chiapas 30700, Mexico; 5 Facultad de Ciencias Biologicas, Universidad Autonoma de Nuevo Leon, Av. Pedro de Alba S/N, Ciudad Universitaria, San Nicolas de los Garza, Nuevo Leon 66455, Mexico; 6 Servicio de Infectologia—Hospital Universitario Dr. Jose Eleuterio Gonzalez, Facultad de Medicina, Universidad Autonoma de Nuevo Leon, Av. Francisco I. Madero and Eduardo Aguirre Pequeño S/N, Mitras Centro, Monterrey, Nuevo Leon 64460, Mexico

**Keywords:** Chikungunya, phylogeny, phylogeography, Mexico

## Abstract

Chikungunya virus (CHIKV) is a mosquito-borne alphavirus that causes Chikungunya fever. CHIKV entered Mexico through the state of Chiapas in October 2014. To fully understand the Chikungunya fever outbreak that occurred in southern Chiapas during 2015, we evaluated 22 PCR-confirmed CHIKV-positive patients, identified CHIKV genetic variability, reconstructed viral dispersal, and assessed possible viral mutations. Viruses were isolated and *E2*, *6K*, and *E1* genes were sequenced. We applied phylogenetic and phylogeographic approaches, modeled mutations, and estimated selective pressure. Different CHIKV strains circulated in Chiapas during summer 2015. Three isolates grouped themselves in a well-supported clade. Estimates show that the outbreak started in Ciudad Hidalgo and posteriorly dispersed towards Tapachula and neighboring municipalities. We found six non-synonymous mutations in our isolates. Two mutations occurred in one isolate and the remaining mutations occurred in single isolates. Mutations E2 T116I and E2 K221R changed the protein surface in contact with the host cell receptors. We could not find positive selected sites in our CHIKV sequences from southern Chiapas. This is the first viral phylogeographic reconstruction in Mexico characterizing the CHIKV outbreak in southern Chiapas.

## 1. Introduction

The Chikungunya virus (CHIKV) is an Alphavirus transmitted by the bite from infected female *Aedes aegypti* and *Aedes albopictus* mosquitoes. It causes an acute febrile disease characterized by joint, head, and muscular pain. It rarely causes death, but joint pain can last for years and cause disability [1,2].

The Chikungunya virus has a 12 kb positive-sense, single-stranded RNA genome which is organized with the nonstructural proteins (nsP1–4) at the 5′ end and the structural proteins (C, E3, E2, 6K, and E1) at the 3′ end [3,4]. The nucleocapsid is enclosed in a lipid envelope derived from the host cell plasma membrane that contains the viral-encoded glycoproteins E1 and E2 [5]. These proteins form heterodimers that are grouped as trimers on the virion surface [6,7]. The E2 glycoprotein on the virion binds to host-cell surface receptors [5]. E1 envelope glycoprotein is primarily responsible for the fusion of viral and cellular membranes within endosomes [8].

Phylogenetic studies have identified three major CHIKV genotypes: West African, East/Central/South African (ECSA), and Asian. The Indian Ocean Linage (IOL), derived from ECSA genotype, arose from La Réunion outbreak in 2006. Viruses from this lineage presented a series of adaptive mutations that mediated enhanced virus transmission by *A. albopictus* [9,10,11]. In the recent American outbreak, the responsible virus belonged to the Asian genotype [1,12].

The first autochthonous Chikungunya fever (CHIKF) case in Mexico was reported in mid-October 2014 at the city of Arriaga, in western Chiapas [13]. Mexican health authorities made the official announcement of confirmed autochthonous transmission on November 7, 2014 [14]. However, CHIKF cases were also detected the first two weeks of October 2014 in Ciudad Hidalgo, in southern Chiapas [15,16]. Confirmed cases continued to appear in Chiapas during November and December 2014. By the first weeks of 2015, CHIKF cases continued in Chiapas and began to appear at the neighboring states of Oaxaca and Guerrero. In Chiapas, during 2015, we can appreciate two waves of CHIKF cases. The first wave appeared from March to April and the second from June to September 2015. Cases began to decrease by the end of the year [17].

Epidemics like rabies, the West Nile virus, Ebola, and Yellow fever have been reconstructed by phylogenetic spatiotemporal analyses to measure the outbreaks’ spatial spread [18,19,20,21,22]. This reconstruction reflects the area than an infected host will explore per unit of time [19]. Beyond phylogenetic analysis, molecular modeling has been used to observe the effects of non-synonymous mutations in CHIKV envelope proteins [23,24,25]. Sahadeo found positive selection in amino acid position 221 within *E2* gene, which is located in the acid-sensitive axis (comprised by residues 210 to 252) [26]. Substitutions in this axis may increase fitness for *Ae. albopictus* infection [10]. 

Chikungunya virus sequences from Southern Chiapas have been used for phylogenetic reconstructions [12,15,27], but have not been analyzed by phylogeographic approaches. The aim of this study was to identify genetic variability, reconstruct viral dispersal, model the non-synonymous mutants, and estimate selection pressure of CHIKV in Southern Chiapas. 

## 2. Materials and Methods 

### 2.1. Study Approval

All subjects gave their informed consent for inclusion before they participated in the study. The study was conducted in accordance with the Declaration of Helsinki, and the protocol was approved by the Ethics Committee from Facultad de Medicina y Hospital Universitario – Universidad Autónoma de Nuevo León with the following registration number: IF12-003 (approval date: 20 August 2012). 

### 2.2. Study Population

We studied 22 PCR-confirmed CHIKV-positive patients from the CHIKV outbreak that occurred in Chiapas, Mexico during 2015. These patients are derived from the previously described Mexican population [27]. Briefly, patients were 18 years old or older, had fever or fever history within the last five days, and one of the following symptoms: polyarthralgia, headache, and/or rash. Patients were enrolled at the secondary-level Hospital Clinic “Dr. Roberto Nettel Flores” in Tapachula, Chiapas, Mexico, from June through July 2015. The particularity of this health clinic is that it only receives State workers and their immediate relatives. At enrollment day, a single serum sample was collected and stored at −70 °C until further use.

### 2.3. Viral RNA Detection

Viral RNA was obtained with QIAamp UltraSens Virus Kit (QIAGEN, Valencia, CA, USA) following manufacturer’s instructions. Screening for CHIKV was carried out using the nsp4 one-step, probe-based, real-time reverse transcription PCR assay (rRT-PCR) described by Lanciotti [28]. The rRT-PCR assays were done using the SuperScript III Platinum One-Step qRT-PCR kit (Invitrogen, Carlsbad, CA) in a 7500 Fast Real-Time PCR System (Applied Biosystems, Carlsbad, CA, USA).

### 2.4. Envelope Genes Amplification and Sequencing

We amplified two different overlapping regions: one composed of 1623 bp that contained partial *6K* and *E1* genes, and another of 1559 bp that contained *E2* and partial *6K* genes. We amplified the remaining *6K* and *E2* genes of the previously ten reported sequences from Chiapas [27]. We used the PrimeScript RT-PCR Kit (TaKaRa, Shiga, Japan) to generate the PCR amplicons. The amplicons were purified using the QIAquick PCR Purification Kit (QIAGEN). Sequencing was performed on a 3500 Genetic Analyzer (Applied Biosystems) using BigDye Terminator v3.1 cycle sequencing kit (Applied Biosystems). Sequences were assembled based on the reference Chikungunya virus sequence from NCBI: NC_004162.2. The sequences were deposited in GenBank under the following accession numbers: MH481881 – MH481900, MK240310 and MK240311. Primers used for amplification and sequencing are described in Table S1.

### 2.5. Data Sets

We retrieved all the available CHIKV sequences from GenBank up to April 2019. We selected the sequences that contained full *E2*, *6K* and *E1* genes. We excluded vaccine strains, synthetic strains, and other non-wild type sequences (Table S2). We included sequences of the three previously described CHIKV genotypes (West Africa, ECSA, and Asian) and the 24 previously reported Mexican CHIKV sequences from Yucatán, northern and southern Chiapas, Jalisco, and Tamaulipas [13,15,29,30]. We combined the 22 newly obtained sequences with the retrieved CHIKV sequences to generate the Complete data set (*n* = 900). Sequences were aligned using the online version of MAFFT v7 [31]. Posteriorly, in MEGA v6.06 (Arizona State University, Tempe, AZ, USA) [32], the alignment was trimmed to leave *E2*, *6K*, and *E1* genes (final length = 2772 bp). We generated two data sets derived from the Complete data set. The Asian data set contained sequences of the Asian genotype, including sequences from the Caribbean outbreak (*n* = 484), and the Chiapas data set which only included sequences isolated in Southern Chiapas, derived from this study and previously reported (*n* = 27) [15]. Nucleotide and amino acid positions are relative to the fragment size.

### 2.6. Phylogenetic Analysis

As a pre-processing step, we screened for sequence recombination using GARD (Genetic Algorithm for Recombination Detection) [33] available in the Datamonkey web server. The evolutionary history was inferred using the maximum-likelihood (ML) method in the IQ-TREE web server [34] and the maximum clade credibility (MCC) in BEAST (Bayesian Evolutionary Analysis Sampling Trees) v1.8.4 (University of Edinburgh, Edinburgh, United Kingdom) [35]. We used the Tamura-Nei substitution model [36] with a discrete Gamma distribution (4 categories) for all the data sets. We evaluated the Complete data set by the ML method and tested phylogeny with 1000 bootstrap replicates using the Ultrafast Bootstrap Approximation [37]. We evaluated the Asian data set with the MCC approach. We included the isolation date, used the relaxed lognormal molecular clock model [38] and the coalescent exponential growth tree prior [39]. The analysis was run for 300 million generations with 10% removed as burn-in. The effective sample size was evaluated with Tracer v1.6 (University of Edinburgh) [40]. The best-supported tree was obtained with TreeAnnotator v1.8.4 (University of Edinburgh), excluding the first 10% of trees as burn-in. 

### 2.7. Phylogeographic Inference

We performed a bayesian continuous phylogeographic analysis on the Chiapas data set using a strict clock model and a coalescent exponential growth tree prior [39] using BEAST v1.8.4 (University of Edinburgh) [35]. First, we inferred the best fitting continuous diffusion process by performing marginal likelihood estimations using path sampling/stepping-stone sampling [41] on the different motion processes (homogenous Brownian model, Cauchy relaxed random walk, and Gamma relaxed random walk models). After a run of 10 million generations, we used 51 path steps on a 10 million chain, sampling every 1000 iterations using a Beta path-step distribution. The Cauchy distribution diffusion rate had the highest log marginal likelihood and was selected for the posterior analysis using a jitter window size of 0.3. The chain was run for 50 million generations and sampling every 5000 steps. The effective sample size was evaluated with Tracer v1.6 (University of Edinburgh) [40]. The summarized MCC tree was obtained with TreeAnnotator v1.8.4 (University of Edinburgh), as previously mentioned. We used SpreaD3 v0.9.6 (Katholieke Universiteit Leuven, Leuven, Belgium) to analyze and visualize CHIKV phylodynamic reconstruction [42]. Shapefiles from Mexico and Chiapas state were downloaded from the Instituto Nacional de Estadística, Geografía e Informática (INEGI) website [43]. Shapefiles were converted to geojson format in ArcGIS v10.2.2 (ESRI, Redlands, CA, USA). The figures were rendered to HTML and KML files for posterior visualization.

### 2.8. Molecular Modeling

We based virtual mutagenesis through molecular on the cryogenic electron microscopy (cryo-EM) structure of Chikungunya E1-E2 Envelope glycoproteins, obtained from the RCSB Protein Data Bank (PDB; ID: 2XFC) [6]. Structural models were visualized on the Swiss PDB viewer software suite (Swiss Institute of Bioinfomatics, Lausanne, Switzerland) [44]. We performed *in silico* mutagenesis and model the protein structure using the SWISS-MODEL modeling server [45]. The mutations were: E2 V113A, E2 T116I, E2 K221R, E1 V4A, and E1 A342V. SWISS-MODEL performed the energy minimization and predicted the structure given the available molecular model. Renders superposing the mutant over the wild-type structures were created using PyMOL OS X v.2.0.5 (Schröedinger, San Diego, CA, USA).

### 2.9. Selection Analyses

We performed selection analyses on the Asian and Chiapas data sets. We used the methods available in the Datamonkey web server, Single-Likelihood Ancestor Counting (SLAC), and Fixed Effects Likelihood (FEL), to identify sites with evidence of positive or negative selection [46]. We looked for episodic diversifying selection using Mixed Effects Model of Evolution (MEME) method [47] and for pervasive diversifying selection using Fast, Unconstrained Bayesian AppRoximation (FUBAR) method [48]. Both are also available in the Datamonkey web server. The *p*-value threshold for SLAC, FEL, and MEME was ≤0.05. The posterior probability threshold for FUBAR was ≥0.95.

## 3. Results

We obtained 22 viral sequences corresponding to the *E2*, *6K*, and *E1* genes. The sequenced viruses were isolated from patients living in different Chiapas’ southern municipalities during the second wave of confirmed CHIKF cases in Chiapas, summer 2015, (Figure S1). Sixteen were isolated from patients living in Tapachula, two from Tuxtla Chico, two from Huixtla, and one from Mazatán and Cacahoatán. Fifty-four percent of the patients were female and the mean age was 37 years old, ranging from 18 to 63 years. All patients had arthralgia, myalgia, and gastrointestinal signs.

The nucleotide and amino acid identity of the studied sequences was 99.6%–100% and 99.7%–100%, respectively. We compared our sequences with an isolate from the Philippines (KU561463) obtained before the Caribbean outbreak. We found twenty-eight synonymous mutations and eight non-synonymous mutations. We detected three synonymous mutations that separated the 2015 Chiapas sequences from the rest of Mexico and the Caribbean. These mutations were transitions and were detected as follows: isolates TA736, HU003 and HU003 contained two mutations in nucleotide positions 735 and 1695 (T → C and C → T, respectively), isolate TA700 contained the mutation in nucleotide position 735 (T → C), and isolates TC010 and TC011 contained the mutation in nucleotide position 1524 (C → T). Two non-synonymous mutations (E2 V368A and 6K L20M) were present in all the strains from the Caribbean outbreak. The remaining six non-synonymous mutations were present in single isolates. Isolate TA689 had two non-synonymous mutations in E2, residues 116 (T → I) and 221 (K → R). Isolates TA750 and TA679 had also mutations in E2, residues 384 (V → A) and 408 (V → I), respectively. Isolates TA702 and TA725 had mutations in E1, residues 4 (V → A) and 342 (A → V), respectively.

When we compared our isolates from 2015 with Chiapas isolates from 2014, we found twenty-seven synonymous mutations and seven non-synonymous mutations. The isolate from northern Chiapas 2014 (KP851709) differed from the southern by two nucleotide changes in *E2* gene, positions 24 and 186. The isolates from southern Chiapas 2014 (KT327163 – KT327167) differed from northern and our isolates from southern Chiapas 2015 by one nucleotide change in *E2* gene, position 338. However, this change caused the additional non-synonymous mutation, E2 V113A. According to nucleotide composition, the genetic distance between Chiapas’ isolates from northern 2014, southern 2014 and southern 2015 is 0.001. According to amino acid composition, the genetic distance between northern and southern Chiapas isolates from 2014 is 0.001. The genetic distance between isolates from northern Chiapas 2014 and southern Chiapas 2015 is 0.00, and between southern 2014 and 2015 isolates is 0.001.

### 3.1. Phylogenetic Analysis and Phylogeographic Inference

We didn’t find recombinant sequences in the pre-processing step and proceeded with the analysis. Maximum-likelihood phylogenetic analysis of the 900 sequences of *E2*, *6K*, and *E1* genes revealed three major genotypes, West African, ECSA, and Asian (Figure S2). Isolates from the Indian Ocean outbreak and Caribbean outbreak form a monophyletic clade in the ECSA and Asian genotypes, respectively. Our isolates from Chiapas 2015 belong to the Asian genotype in the Caribbean outbreak monophyletic clade.

**Figure 1:**
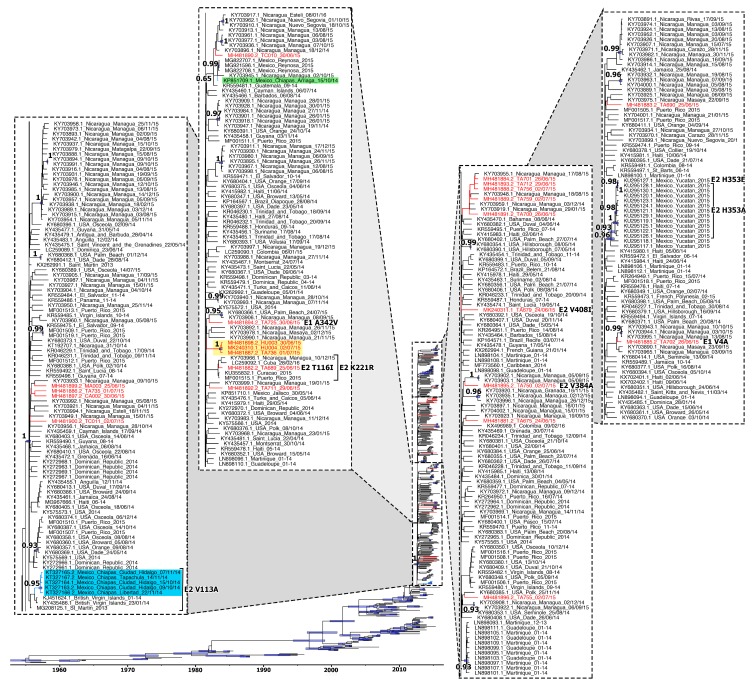
Maximum clade credibility phylogeny of Chikungunya virus (CHIKV) Asian genotype. Taxon labels include accession number, isolation place, and year. The Caribbean outbreak clade was split in four parts and magnified. Branch lengths are scaled to the sampling and divergence times. Node heights with a posterior probability of >90 have thick blue horizontal node bars that represent 95% higher posterior density (HPD) values. Posterior probabilities are indicated as bold numbers. Relevant amino acid changes are indicated at the tips. The sequences obtained in this study are marked in red. Sequences from southern Chiapas in 2014 are highlighted in blue; while the sequence from northern Chiapas in 2014 is highlighted in green. The Huixtla clade is highlighted in yellow.

We separately analyzed the Asian genotype by the maximum clade credibility approach to better understand the dynamics and relationship of our sequences with the sequences of the Caribbean outbreak. The estimated substitution rates for the Asian genotype was 6.7 × 10^−4^ substitutions per site per year (95% higher posterior density (HPD): 5.6–7.9 × 10^−4^ subs/site/yr.). The estimated time to the most recent common ancestor (tMRCA) of the Asian genotype was December 1954, with a 95% HPD between December 1951 and April 1957. As it was reported before [12,25], the Asian genotype circulated in South and Southeast Asia in the 1950s and 1960s. These viruses continued spreading though Southeast Asia, the South Pacific Islands, and finally to the Caribbean. Sequences from the Caribbean outbreak created their own clade with high supported values posterior probability (PP) ≥0.99 (Figure 1). The estimated tMRCA for the Caribbean clade was July 2012 (95% HPD: March–November 2012). Isolates from southern Chiapas in 2014 grouped together in a well-supported clade (PP 0.95), closely related to isolates from British Virgin Islands (Figure 1). The isolate from northern Chiapas in 2014 grouped together with isolates from Guatemala (2014), Nicaragua (2015), and the Mexican city of Reynosa, Tamaulipas (2015) in a moderate-supported clade. The estimated tMRCA for the southern and northern Chiapas 2014 clusters were July 2014 (95% HPD: April–October 2014 and 95% HPD: April–August 2014, respectively).

**Figure 2:**
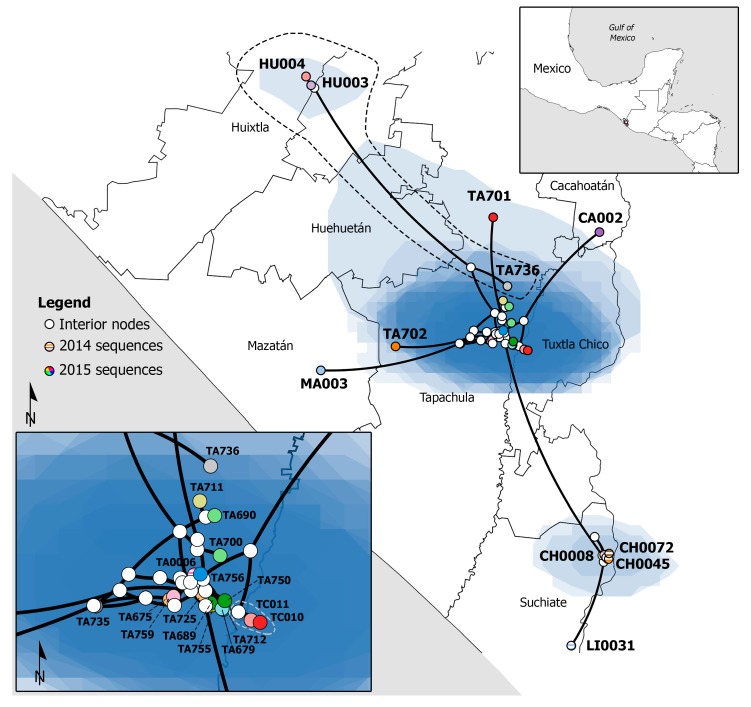
Spatiotemporal epidemic history of CHIKV in Southern Chiapas. White circles represent interior nodes, while colored circles represent the analyzed sequences. Circles with horizontal lines correspond to sequences from 2014. Circles with solid colors correspond to sequences from 2015. The blue polygons represent the 80% HPD of the location. The black dashed lined shows the Huixtla clade. The gray dashed line shows the Tuxtla Chico clade. The upper right square shows the location of Southern Chiapas. The lower left square is a magnification of the sequences from Tapachula and Tuxtla Chico. The outbreak started in Ciudad Hidalgo and spread upwards to Tapachula. Sequences are named after the isolation place. HU: Huixtla; TA: Tapachula; CA: Cacahoatán; MA: Mazatán; TC: Tuxtla Chico; CH: Ciudad Hidalgo; LI: La Libertad.

Considering only sequences from southern Chiapas, the spatiotemporal analysis estimation places the root of the tree at Ciudad Hidalgo (Suchiate, Chiapas), 1 km west from the Guatemalan city Ciudad Tecún Umán. The estimated tMRCA of the root was September 2014 (95% HPD: July–October 2014). The virus spread through Ciudad Hidalgo during October and November and spread to the neighboring community, La Libertad (Figure 2). By mid-November, CHIKV was identified in the city of Tapachula, 27 km northwest from the estimated tree root. The estimated MRCA of all Tapachula’s viruses was placed in the Southeast suburb of Tapachula called San José El Edén in November 2014 (95% HPD: October–November 2014). The virus continued circulating in Tapachula during the rest of 2014 and start of 2015.

None of the newly obtained sequences from southern Chiapas in 2015 grouped with sequences from northern or southern Chiapas isolated in 2014. Isolates HU003, HU004, and TA736 were the only ones grouped in a well-supported clade that we denominated Huixtla clade (Figure 1). The sequence of five isolates from Tapachula (TA701, TA712, TA756, TA759, and TA700) grouped with sequences from Nicaragua, one isolated in 2014 and three isolated in 2015, however, this clade was low supported (Figure 1). Isolate TA789 grouped with isolates from Nicaragua 2015, Curacao 2015, Puerto Rico 2015, and Cuba 2016. This clade had low support according to MCC analysis and moderate support according to ML analysis (PP 0.56 and bootstrap 77, respectively) (Figure S2). Isolate TA702 grouped with viruses isolated from Nicaragua in 2015 in a moderately supported clade (Figure 1). The remaining isolates scattered through the phylogenetic tree.

According to the spatiotemporal analysis, the virus spread from Tapachula to Huixtla, Tuxtla Chico, Cacahoatán, and Mazatán (Figure 2). The MRCA of Huixtla clade was estimated to exist in April 2015 (95% HPD: February–June 2015), 7 km northwest of Downtown Tapachula. Samples isolated from Tuxtla Chico only grouped together in the ML tree with moderate support (Figure S2). The MRCA of the Tuxtla Chico clade was estimated to exist in May 2015 (95% HPD: February–June 2015) at San Antonio Cahoacán, a suburban area of East Tapachula. We estimated that virus moved, on average 160 m/day (95% HPD: 82–284 m/day). The location of two isolates was incorrectly estimated (TA701 and TA702). These viruses were isolated from the same household at North Tapachula. The estimated location of isolate TA701 was 8 km north, and TA702 was 14 km southwest of the real location.

Viruses isolated in other parts from Mexico (Tamaulipas and Yucatán) formed well-supported clades (Figure 1). The isolate from Jalisco, which corresponded to an imported case, didn’t group with others. Isolates from Nicaragua, Puerto Rico, Dominican Republic, Guadalupe, and Martinique formed well-supported clades, as previously reported [25,49].

### 3.2. Molecular Modeling

**Figure 3:**
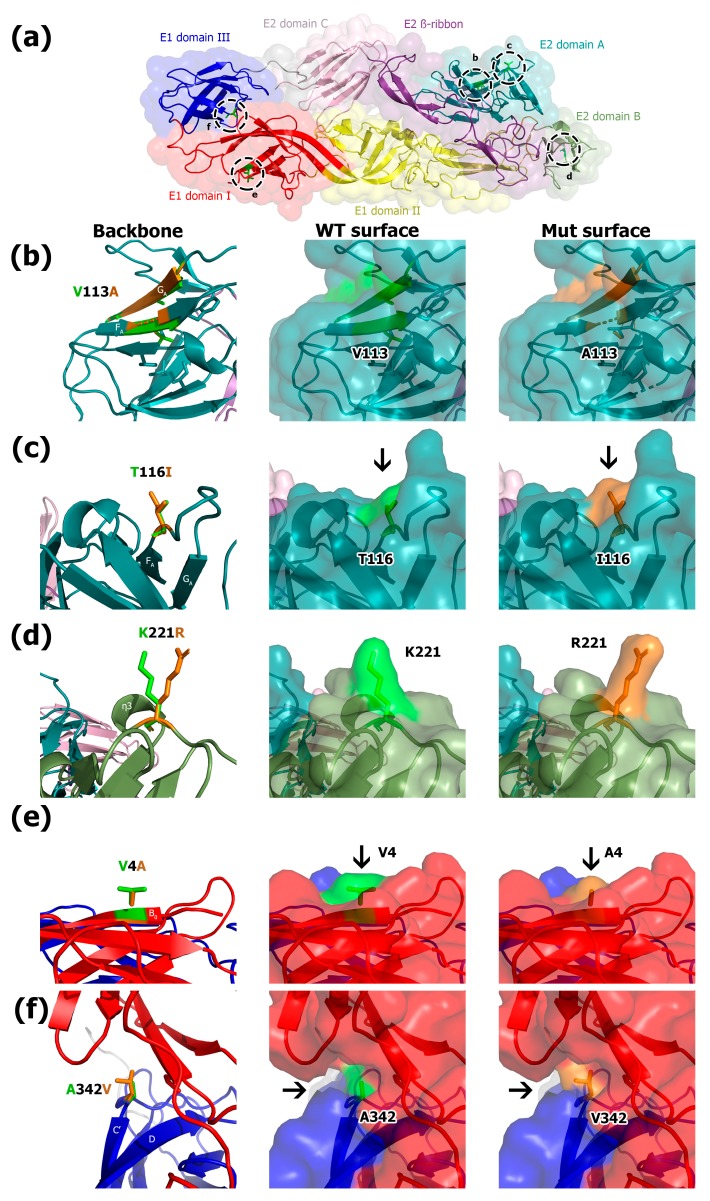
Molecular model of CHIKV envelope proteins. (**a**) Structure of E1 and E2 CHIKV proteins. E1 domains I, II, and III are shown in red, yellow and blue, respectively. E2 domain A is colored teal, B dark green, C pink, and ß-ribbon dark purple. The five non-synonymous mutations are shown in a discontinuous black circle. (**b**) E2 V113A mutation present in isolates from Southern Chiapas in 2014. (**c**) E2 T116I mutation present in isolate TA689. (**d**) E2 K221R mutation present in isolate TA689. (**e**) E1 V4A mutation present in isolate TA702. (**f**) E1 A342V mutation present in isolate TA725. The wild-type (WT) amino acid is colored lime, and the mutated amino acid is colored orange. The left panel shows the protein backbone. Central and right panels show the protein surface of the WT amino acid and the mutation, respectively. Strand and helixes are indicated in white. Black arrows indicate the change in the protein surface.

Based on the available cryo-EM structure for Chikungunya E1/E2 protein (PDB ID: 2XFC), we introduced *in silico* single amino acid residue substitutions and performed molecular modeling through energy minimization and fitting to the available structure. We modeled five of the seven non-synonymous mutations, three mutations in E2 protein (A113V, T116I, and K221R) and two mutations in E1 protein (V4A and A342V) (Figure 3a). The E2 protein mutations V384A and V408I occurred at the transmembrane segment and the cytoplasmic tail respectively, and could not be modeled.

The E2 A113V mutation occurred only in the southern Chiapas’ sequences from 2014. The mutation is located at the end of domain A, specifically at F_A_ strand. This mutation interrupts the ß sheet and slightly deviates strands F_A_ and G_A_. The side chain of residue E2 113 faces towards the hydrophobic core of the protein and did not noticeably alter the overall structure of the protein or the solvent-accesible surface (Figure 3b).

The E2 T116I mutation also occurred at the end of domain A. This mutation is located after the F_A_ strand at the FG loop. This substitution changes the surface, because these side chains are orientated towards the solvent accessible surface and the bulkier side chain of isoleucine creates a larger protrusion into the solvent (Figure 3c). This mutation changes a polar residue (threonine) with a hydrophobic residue (isoleucine).

The E2 K221R mutation occurred at the end of domain B. This mutation is located in the EF loop before the η^3^ helix at the end of the domain. This mutation generates a remarkable change in the solvent-accessible surface of the protein, which is in contact with the host cell receptors. Although both residues (lysine and arginine) have a positive charge, arginine’s bulkier side chain is located at the opposite direction of lysine’s side chain (Figure 3d).

The E1 V4A mutation occurred at the beginning of domain I, specifically at B_0_ strand. This mutation alters the protein surface in contact with the viral membrane. The smaller methyl group of alanine in the mutation creates a smaller protrusion on the surface of the protein than the larger valine residue, altering the solvent-accessible surface of the protein (Figure 3e).

The E1 A342V mutation occurred in domain III. This mutation is located in the DC’ loop. Valine’s bulkier side-chain decreased the depth of the pocket within the hinge region of the envelope protein (Figure 3f). This mutation also changes the solvent-accessible surface.

### 3.3. Selection Analyses

We tested our partial sequences for positive and negative selection, episodic diversifying selection and pervasive diversifying selection. The SLAC method only detected sites under negative selection (Table S3). We found evidence of episodic diversifying selection and pervasive diversifying selection in two sites at the *E2* gene (Table 1).

In the Asian data set, FEL, FUBAR, and MEME identified codon 221 as positively selected. The amino acid changes detected in residue E2 221 were: lysine to glycine and lysine to arginine. The first mutation (K → G) was detected in a sequence from Thailand dated to 1958. The second mutation (K → R) was detected in eight sequences: two from Southeast Asia, before the Caribbean outbreak, and six from the Caribbean, North and Central America, during the Caribbean outbreak.

MEME also identified codon 353 as positively selected. The amino acid changes detected in residue E2 353 were: histidine to alanine and histidine to glutamate. These mutations were detected in the viral sequences isolated in Yucatán 2015. The first mutation (H → A) was detected in 11 of 14 Yucatán sequences, and the second mutation (H → E) in 1 of 14 sequences. In the Chiapas data set, FUBAR only detected sites under negative selection (Table S3).

## 4. Discussion

We obtained twenty-two *E2*, *6K*, and *E1* CHIKV sequences from the southern Chiapas’ municipalities during June–July 2015. At the time of writing, Yucatán was the Mexican state with most reported CHIKV sequences (14) [29]. The sequences generated in this study now make Chiapas the state with most reported CHIKV sequences. This facilitates further analyses of CHIKV evolution through Mexico. We didn’t find the mutation E1 A226V for enhanced infection of *A. albopictus* [9].

Our estimated substitution rates for the Asian genotype was greater than the previously reported [25,26,50,51,52]. An explanation is that we only used *E2*, *6K*, and *E1* genes for the estimation. In contrast with previous reports which estimated substitution rates using the whole genome. Another reason is that we analyzed 484 sequences of the Asian genotype, almost double of the bigger studies [25]. As previously mentioned, we estimated that the tMRCA of the Asian genotype was December 1954. Our estimate agrees with the year estimated by Chen and colleagues (March 1954), who used whole genomes in their analysis, [12]. Other estimates ranged from 1953 to November 1957 [25,51]. In our analysis, July 2012 was the estimated tMRCA of the Caribbean clade, agreeing with Chen and colleagues report [12]. Other tMRCA estimates ranged from November 2009 to March 2013 [25,26,52].

Phylogenetic analyses place our isolates at the Caribbean outbreak clade, but not grouped with sequences from southern Chiapas isolated in 2014. Sequences isolated in southern Chiapas in 2014 [15] contained the non-synonymous mutation E2 V113A, as previously reported by Sahadeo [26]. We only observed this mutation in sequences from southern Chiapas and British Virgin Islands [53]. We hypothesize that Chiapas had two different CHIKV introductions during this outbreak, from Guatemala and the Caribbean. Tan and colleagues’ CHIKV whole genome analysis supports this hypothesis [25]. They found that isolates from southern Chiapas in 2014 grouped in a well-supported clade they named CO4 and the isolate from northern Chiapas in 2014 grouped with other isolates from Nicaragua and Caribbean in another well-supported clade they named CO1 (specifically clade CO1.4). The change in nucleotide position 338 leading to the E2 V113A mutation in southern Chiapas sequences can be explained by genetic drift or sequencing error. It is possible that Chiapas had a third CHIKV introduction during 2015, this time from Nicaragua, since five sequences from Tapachula grouped with sequences from Nicaragua obtained during 2014 and 2015. Further viral sequencing is needed to confirm it. We need to obtain the sequence of the whole genome to confirm the similarity of the sequences and obtain a well-supported clade.

The mean tMRCA of the southern Chiapas isolates in 2014 estimated by phylogenetic reconstruction differs from the spatiotemporal reconstruction by two months. Nevertheless, the spatiotemporal estimate is inside the 95% higher posterior density of the phylogenetic reconstruction. This difference can be attributed to the number of sequences involved in the analysis and that spatiotemporal reconstruction considered also location. Both reconstructions, phylogenetic and spatiotemporal, consider October in the estimated tMRCA. This agrees with the large number of patients reporting febrile illness accompanied by rash and unusual arthralgia in Ciudad Hidalgo Chiapas during October 2014 [15].

According to the phylogenetic analysis, the virus isolated in northern Chiapas in 2014 moved through Mexico until reaching the northern state of Tamaulipas in 2015. The fate of the viruses isolated in southern Chiapas during 2014 is unknown. A fitter virus probably replaced them during the first wave of CHIKF cases in 2015, however, this should be confirmed by sequencing viruses from stored serum samples. Isolate TA689 grouped with the other four isolates that shared the E2 K221R mutation. We couldn’t obtain the exact isolation date from two isolates, Curacao and Puerto Rico, therefore, we can infer that viruses from those two places reached Chiapas in June 2015. Viruses with that mutation continued circulating in the Caribbean and were detected in Nicaragua and Cuba.

This is the first spatiotemporal reconstruction of CHIKV in Mexico. We can trace back how the virus spread through Chiapas. This studies help to predict the extent that CHIKV can spread. Chikungunya virus spread from the south border city of Ciudad Hidalgo to La Libertad and Tapachula. The virus circulated in Tapachula and posteriorly spread to neighboring municipalities, Mazatán, Cacahoatán, Tuxtla Chico and Huixtla. We estimated that virus moved, on average 160 m/day (95% HPD: 82–284 m/day). In contrast, a report from Carabobo state in Venezuela, using epidemiological data, estimated that virus spread on average 82.9 m/day [54]. One reason for the inconsistency is that in Venezuela they used the information of 735 cases, instead of 27. Isolates TA701 and TA702 were probably misplaced because they differed in four nucleotide positions, one translating as the non-synonymous mutation E1 V4A. The phylogeographic reconstruction considered these changes and estimated a different location. It is important to note that even though isolates TA701 and TA702 were obtained from patients sharing the same household, the sequences didn’t group together in the phylogenies estimated by ML or MCC approaches.

Only mutations E2 A113V and E2 K221R were present in more than one of the studied sequences. The molecular modeling of mutation A113V, present in isolates from Southern Chiapas and British Virgin Islands in 2014, showed that the overall structure of E2 protein is conserved. The mutation E2 K221R present in isolates from Thailand, Philippines, Nicaragua, Puerto Rico, Curacao, Cuba and Chiapas alters the protein surface of domain B that is in contact with the host cell surface receptors. Mutations in the E_B_-η^3^ loop in Ross River virus makes it able to bind to heparan sulfate [55]. Therefore, this mutation should be further studied. The other mutations were only present in one isolate from Chiapas. We hypothesize that these mutations were posteriorly removed by purifying selection. None of E2 and E1 mutations occurred in the transitional epitopes [56,57].

We found six non-synonymous mutations in our Chiapas 2015 sequences, four in E2, and two in E1. Only the site of one non-synonymous mutation, E2 221, had evidence of episodic and pervasive diversifying selection. As previously mentioned, glycine is present in the sequence from Thailand 1958 and arginine in the sequences from Thailand 1996, Philippines 2011, Curacao 215, Nicaragua 2015, Puerto Rico 2015, Chiapas 2015, and Cuba 2016. The positive selection is probably explained by the mutation present in the isolate from Thailand 1958, because the site was not predicted to be under positive selection when the Chiapas data set was analyzed. This site was previously reported as positively selected [26]. As previously shown, MEME found evidence of positive selection at site 353. Mutations at this site were detected in sequences from Yucatán state. Mutation H353A changes one of the conserved histidines in the previously unidentified D-loop in E2 [7]. Mutation of this residue causes budding defect and decreases virus particle stability [58]. Posterior mutations in the D-loop, for example I356Y, can alter the correct functions of E2/E1 lattice during budding [58]. The effect of these mutations in Yucatán viruses has yet to be determined.

Our study has several limitations: we only sampled during summer 2015, we only sampled patients from a single hospital, and only sequenced a section of the structural protein genes. Sampling only in one period we miss detecting the viruses that could prevail in the population. Sampling from a single hospital debilitates the strength of the study because it doesn’t represent the general population and could not accurately explain viral distribution. Sequencing only a fragment of structural protein genes impairs the resolution of the phylogenetic tree. More information would make the phylogenetic and phylogeographic inferences more accurate. Having the whole genome, we could distinguish if isolates from different countries and dates really cluster together in a clade.

## 5. Conclusions

We found different CHIKV strains that were circulating at the same time in Southern Chiapas during summer 2015. We found no positive selection in the studied genes in the circulating viruses from Chiapas.

## Figures and Tables

**Table 1:** Positive selected sites in *E2* gene in the studied CHIKV Asian lineages ^a^.

Method	Data Set	Codon Position	Significance	Mutation	Sequences
SLAC	Asian	-	-	-	-
	Chiapas	-	-	-	-
FEL	Asian	221	0.031 ^b^	K → G	Thailand 1958 (HM045810)
				K → R	Thailand 1996 (KX262987)
					Philippines 2011 (KU561459)
					Curacao 2015 (KU355832)
					Nicaragua 2015 (KY703948)
					Nicaragua 2015 (KY703966)
					Puerto Rico 2015 (MF001515)
					TA689 2015 (MH481882)
					Cuba 2016 (LC259092)
	Chiapas	-	-	-	-
FUBAR	Asian	221	0.991 ^c^	K → G	Thailand 1958 (HM045810)
				K → R	Thailand 1996 (KX262987)
					Philippines 2011 (KU561459)
					Curacao 2015 (KU355832)
					Nicaragua 2015 (KY703948)
					Nicaragua 2015 (KY703966)
					Puerto Rico 2015 (MF001515)
					TA689 2015 (MH481882)
					Cuba 2016 (LC259092)
	Chiapas	-	-	-	-
MEME	Asian	221	0.01 ^b^	K → G	Thailand 1958 (HM045810)
				K → R	Thailand 1996 (KX262987)
					Philippines 2011 (KU561459)
					Curacao 2015 (KU355832)
					Nicaragua 2015 (KY703948)
					Nicaragua 2015 (KY703966)
					Puerto Rico 2015 (MF001515)
					TA689 2015 (MH481882)
					Cuba 2016 (LC259092)
		353	<0.001 ^b^	H → A	Yucatán 2015 (KU295119 – 26, 28–30)
				H → E	Yucatán 2015 (KU295127)
	Chiapas	-	-	-	-

^a^ Codon positions correspond to *E2* gene. Hyphens indicate lack of information. SLAC, single-likelihood ancestor counting; FEL, fixed effects likelihood; FUBAR, fast unconstrained bayesian approximation; MEME, mixed effects model of evolution; K, lysine; G, glycine; R, arginine; H, histidine; A, alanine; E, glutamate. ^b^ *p*-value. ^c^ posterior probability.

## Supplementary Materials

The following are available online at https://www.mdpi.com/1999-4915/11/8/714/s1, Figure S1: Chikungunya fever outbreak in Chiapas, epidemic curve by epidemiological weeks; Figure S2: Maximum-likelihood phylogeny based on the complete set of *E2*, *6K* and *E1* genes of 900 CHIKV sequences; Table S1: Primers used for amplification and sequencing of CHIKV genes; Table S2: Asian genotype sequences used in this study; Table S3: Negative selected sites within *E2*, *6K* and *E1* genes; Interactive Map 1: Distribution of CHIKV in southern Chiapas.
