# Supplementary material for: Genetic Variability of Chikungunya Virus in Southern Mexico
Source: Viruses. 2019 Aug 5;11(8):714. doi: 10.3390/v11080714 (PMC6722872; doi:10.3390/v11080714)
Supplement: Supplementary file 1 [file viruses-11-00714-s001.zip › Suplementary/Supplementary Table 1.docx]

**Table S1.** Primers used for amplification and sequencing of envelope genes ^a^.

| **Gene** | **Primer** | **Sequence** | **5’ Position** | **Fragment size (bp)** |
| --- | --- | --- | --- | --- |
| E1 | E15F | ACACCGTACGAACTGACACC | 9745 | 1623 |
|  | E13R | GTCTCTTTAGGGACGCGTATG | 11357 |  |
|  | E1CFs | AGCGAAGCACATGTGGAGAA | 10305 |  |
|  | E1CRs | CGTCCGGTATGTCGATGGAG | 10848 |  |
| E2 | E25F | CACACCCTGCTGCTACGAAA | 8417 | 1559 |
|  | E23R | CGACGCTCAGTACGGCTAAA | 9975 |  |
|  | E2CFs | GGAGAAACTCTGACGGTGGG | 8865 |  |
|  | E2CRs | CGGCACGGTTAACCTGATCT | 9509 |  |

^a^ Primers indicated with “s” were used for sequencing. The 5’ position is according to Chikungunya isolate AMA2798/H804298, Genbank accession number: KP164567.1
