# Supplementary material for: Genetic Variability of Chikungunya Virus in Southern Mexico
Source: Viruses. 2019 Aug 5;11(8):714. doi: 10.3390/v11080714 (PMC6722872; doi:10.3390/v11080714)
Supplement: Supplementary file 1 [file viruses-11-00714-s001.zip › Suplementary/Supplementary Table 2.docx]

**Table S2.** Asian genotype sequences used in this study.

| **Name** | **Accession number** | **Strain name** | **Isolation date** |
| --- | --- | --- | --- |
| India_Kolkata | EF027140.1 | IND-63-WB1 | 1963 |
| India_Barsi | EF027141.1 | IND-73-MH5 | 1973 |
| Indonesia | EU192143.1 | 0706aTw | 2007 |
| Malaysia_Bagan_Panchor | EU703759.1 | MY002IMR/06/BP | 2006 |
| Malaysia_Bagan_Panchor | EU703760.1 | MY003IMR/06/BP | 2006 |
| Malaysia_Bagan_Panchor | EU703761.1 | MY019IMR/06/BP | 2006 |
| Malaysia_Bagan_Panchor | EU703762.1 | MY021IMR/06/BP | 2006 |
| Indonesia | FJ807886.1 | 0712aTw | 2007 |
| Indonesia | FJ807887.1 | 0712bTw | 2007 |
| Indonesia | FJ807888.1 | 0802aTw | 2008 |
| Indonesia | FJ807889.1 | 0804aTw | 2008 |
| Indonesia | FJ807890.1 | 0806aTw | 2008 |
| Indonesia | FJ807891.1 | 0811aTw | 2008 |
| Indonesia | FJ807897.1 | 0706aTw | 2007 |
| Malaysia_Perak | FN295483.3 | MY/06/37348 | 03/2006 |
| Malaysia_Perak | FN295484.2 | MY/06/37350 | 03/2006 |
| France | FR846307.1 | Pt11352 | 03/07/2009 |
| New_Caledonia | HE806461.1 | NC/2011-568 | 28/02/2011 |
| Thailand | HM045787.1 | SV0444-95 | 1995 |
| India | HM045788.1 | PO731460 | 1973 |
| Thailand | HM045789.1 | 6441-88 | 1988 |
| Philippines | HM045790.1 | PhH15483 | 17/07/1985 |
| Indonesia | HM045791.1 | JKT23574 | 1983 |
| Thailand | HM045796.1 | CO392-95 | 1995 |
| Indonesia | HM045797.1 | RSU1 | 1985 |
| Philippines | HM045800.1 | Hu/85/NR/001 | 1985 |
| Thailand | HM045802.1 | K0146-95 | 1995 |
| India | HM045803.1 | I-634029 | 06/11/1963 |
| Thailand | HM045808.1 | 3412-78 | 1978 |
| Thailand | HM045810.1 | TH35 | 1958 |
| India | HM045813.1 | Gibbs 63-263 | 06/11/1963 |
| Thailand | HM045814.1 | 1455-75 | 1975 |
| China | KC488650.1 | CHIKV-JC2012 | 2012 |
| Indonesia | KC879559.1 | 2001908323-BDG E1 | 2001 |
| Indonesia | KC879560.1 | 2001907981-BDG E1 | 2001 |
| Indonesia | KC879561.1 | 2001918633-BDG E1 | 2001 |
| Indonesia | KC879562.1 | 2004904899-BDG E1 | 2004 |
| Indonesia | KC879563.1 | 2004906033-BDG E1 | 2004 |
| Indonesia | KC879564.1 | 2004906079-BDG E1 | 2004 |
| Indonesia | KC879565.1 | 2004904879-BDG E1 | 2004 |
| Indonesia | KC879566.1 | 2003909662-BDG E1 | 2003 |
| Indonesia | KC879567.1 | 2003910438-BDG E1 | 2003 |
| Indonesia | KC879568.1 | 2002918310-BDG E1 | 2002 |
| Indonesia | KC879569.1 | 2002918314-BDG E1 | 2002 |
| Indonesia | KC879570.1 | 2003902452-BDG E1 | 2003 |
| Indonesia | KC879571.1 | 2003902453-BDG E1 | 2003 |
| Indonesia | KC879572.1 | 2003902459-BDG E1 | 2003 |
| China_Zheijian | KF318729.1 | chik-sy | 06/07/2012 |
| Rusia_Indonesia | KF872195.1 | LEIV-CHIKV/Moscow/1/2013 | 24/09/2013 |
| Micronesia_Yap | KJ451622.1 | 3807 | 10/2013 |
| Micronesia_Yap | KJ451623.1 | 3462 | 10/2013 |
| British_Virgin_Islands | KJ451624.1 | 99659 | 01/2014 |
| Micronesia_Yap | KJ689452.1 | Yap 13-2039 | 11/2013 |
| Micronesia_Yap | KJ689453.1 | Yap 13-2148 | 11/2013 |
| Indonesia_Bali | KM673291.1 | DH130003 | 01/2013 |
| Malaysia | KM923917.1 | M125 | 09/03/2007 |
| Malaysia | KM923918.1 | M127 | 09/03/2007 |
| Malaysia | KM923919.1 | M128 | 09/03/2007 |
| Malaysia | KM923920.1 | M129 | 09/03/2007 |
| Brazil_Oiapoque | KP164567.1 | AMA2798/H804298 | 28/08/2014 |
| Brazil_Recife | KP164571.1 | PER160/H803609 | 03/07/2014 |
| Brazil_Belem | KP164572.1 | TR206/H804187 | 21/08/2014 |
| Mexico_Chiapas_Arriaga | KP851709.1 | InDRE 51CHIK | 15/10/2014 |
| Mexico_Jalisco | KP851710.1 | InDRE 4CHIK | 30/05/2014 |
| Trinidad_and_Tobago | KR046227.1 | VE53_20 | 30/08/2014 |
| Trinidad_and_Tobago | KR046228.1 | VE54_19 | 11/09/2014 |
| Trinidad_and_Tobago | KR046229.1 | VE56_9 | 17/09/2014 |
| Trinidad_and_Tobago | KR046230.1 | VE56_13 | 18/09/2014 |
| Trinidad_and_Tobago | KR046231.1 | VE54_20 | 09/11/2014 |
| Trinidad_and_Tobago | KR046232.1 | VE56_20 | 20/09/2014 |
| Trinidad_and_Tobago | KR046233.1 | VE57_2 | 20/09/2014 |
| Trinidad_and_Tobago | KR046234.1 | VE55_4 | 12/09/2014 |
| Puerto_Rico | KR264949.1 | PR-S4 | 15/07/2014 |
| Puerto_Rico | KR264950.1 | PR-S5 | 16/07/2014 |
| Puerto_Rico | KR264951.1 | PR-S6 | 14/08/2014 |
| Puerto_Rico | KR559470.1 | WHCHK1 | 11/2014 |
| El_Salvador | KR559471.1 | WHCHK2 | 10/2014 |
| El_Salvador | KR559472.1 | WHCHK3 | 06/2014 |
| French_Polynesia | KR559473.1 | WHCHK4 | 02/2015 |
| Puerto_Rico | KR559474.1 | WHCHK5 | 09/2014 |
| El_Salvador | KR559475.1 | WHCHK6 | 09/2014 |
| Haiti | KR559476.1 | WHCHK7 | 07/2014 |
| Dominican_Republic | KR559477.1 | WHCHK8 | 07/2014 |
| Haiti | KR559478.1 | WHCHK9 | 05/2014 |
| Dominica_Republic | KR559479.1 | WHCHK10 | 04/2014 |
| Virgin_Islands | KR559480.1 | WHCHK11 | 09/2014 |
| Guatemala | KR559481.1 | WHCHK12 | 09/2014 |
| Virgin_Islands | KR559482.1 | WHCHK13 | 08/2014 |
| Puerto_Rico | KR559483.1 | WHCHK14 | 10/2014 |
| El_Salvador | KR559484.1 | WHCHK15 | 11/2014 |
| Virgin_Islands | KR559485.1 | WHCHK16 | 10/2014 |
| Panama | KR559486.1 | WHCHK17 | 11/2014 |
| Honduras | KR559487.1 | WHCHK18 | 07/2014 |
| Honduras | KR559488.1 | WHCHK19 | 09/2014 |
| Jamaica | KR559489.1 | WHCHK20 | 10/2014 |
| Guyana | KR559490.1 | WHCHK21 | 08/2014 |
| Colombia | KR559491.1 | WHCHK22 | 08/2014 |
| Saint_Lucia | KR559492.1 | WHCHK23 | 08/2014 |
| American_Samoa | KR559493.1 | WHCHK24 | 2014 |
| Virgin_Islands | KR559494.1 | WHCHK25 | 07/2014 |
| Puerto_Rico | KR559495.1 | WHCHK26 | 07/2014 |
| Guyana | KR559496.1 | WHCHK27 | 07/2014 |
| St_Barts | KR559497.1 | WHCHK28 | 06/2014 |
| Dominican_Republic | KR559498.1 | WHCHK29 | 03/2014 |
| Nicaragua | KT192707.1 | 11540 | 31/10/2014 |
| Philippines | KT308159.1 | CPCC007800Y01 | 2012 |
| Philippines | KT308160.1 | CPCC017800Y01 | 2012 |
| Philippines | KT308161.1 | CPCC065200Y01 | 2012 |
| Philippines | KT308162.1 | CPCC083400Y01 | 2012 |
| Philippines | KT308163.1 | CPCC095700Y01 | 2012 |
| Mexico_Chiapas_Ciudad_Hidalgo | KT327163.2 | CH0008 | 09/10/2014 |
| Mexico_Chiapas_Ciudad_Hidalgo | KT327164.1 | CH0045 | 15/10/2014 |
| Mexico_Chiapas_Ciudad_Hidalgo | KT327165.2 | CH0072 | 07/11/2014 |
| Mexico_Chiapas_Libertad | KT327166.2 | LI0031 | 22/11/2014 |
| Mexico_Chiapas_Tapachula | KT327167.2 | TA0006 | 14/11/2014 |
| Mexico_Yucatan | KU295117.1 | YUC-M21 | 2015 |
| Mexico_Yucatan | KU295118.1 | YUC-M16 | 2015 |
| Mexico_Yucatan | KU295119.1 | YUC-A43 | 2015 |
| Mexico_Yucatan | KU295120.1 | YUC-A40 | 2015 |
| Mexico_Yucatan | KU295121.1 | YUC-A36 | 2015 |
| Mexico_Yucatan | KU295122.1 | YUC-A26 | 2015 |
| Mexico_Yucatan | KU295123.1 | YUC-A22 | 2015 |
| Mexico_Yucatan | KU295124.1 | YUC-A21 | 2015 |
| Mexico_Yucatan | KU295125.1 | YUC-A39 | 2015 |
| Mexico_Yucatan | KU295126.1 | YUC-A33 | 2015 |
| Mexico_Yucatan | KU295127.1 | YUC-A30 | 2015 |
| Mexico_Yucatan | KU295128.1 | YUC-A15 | 2015 |
| Mexico_Yucatan | KU295129.1 | YUC-A13 | 2015 |
| Mexico_Yucatan | KU295130.1 | YUC-A7 | 2015 |
| Brazil | KU355832.1 | RJ/CHIKV/2015 | 2015 |
| Indonesia | KU561436.1 | 0904bTw | 2009 |
| Indonesia | KU561437.1 | 0908aTw | 2009 |
| Indonesia | KU561438.1 | 0909aTw | 2009 |
| Indonesia | KU561439.1 | 1002bTw | 2010 |
| Indonesia | KU561440.1 | 1005aTw | 2010 |
| Indonesia | KU561441.1 | 1005bTw | 2010 |
| Indonesia | KU561442.1 | 1006bTw | 2010 |
| Indonesia | KU561443.1 | 1011aTw | 2010 |
| Indonesia | KU561444.1 | 1207aTw | 2012 |
| Indonesia | KU561445.1 | 1301aTw | 2013 |
| Indonesia | KU561446.1 | 1302aTw | 2013 |
| Indonesia | KU561447.1 | 1303aTw | 2013 |
| Indonesia | KU561448.1 | 1304aTw | 2013 |
| Indonesia | KU561449.1 | 1307aTw | 2013 |
| Indonesia | KU561450.1 | 1308bTw | 2013 |
| Indonesia | KU561451.1 | 1308cTw | 2013 |
| Indonesia | KU561452.1 | 1310aTw | 2013 |
| Indonesia | KU561453.1 | 1312cTw | 2013 |
| Indonesia | KU561454.1 | 1403aTw | 2014 |
| Indonesia | KU561455.1 | 1403bTw | 2014 |
| Indonesia | KU561456.1 | 1404aTw | 2014 |
| Indonesia | KU561457.1 | 1406aTw | 2014 |
| Indonesia | KU561458.1 | 1408aTw | 2014 |
| Philippines | KU561459.1 | 1108aTw | 2011 |
| Philippines | KU561460.1 | 1307bTw | 2013 |
| Philippines | KU561461.1 | 1308aTw | 2013 |
| Philippines | KU561462.1 | 1309aTw | 2013 |
| Philippines | KU561463.1 | 1312aTw | 2013 |
| Philippines | KU561464.1 | 1312bTw | 2013 |
| Philippines | KU561465.1 | 1401aTw | 2014 |
| Singapore | KU561466.1 | 1307cTw | 2013 |
| Indonesia | KX097982.1 | JMB-154 | 2015 |
| Indonesia | KX097986.1 | JMB-192 | 2015 |
| Indonesia | KX097988.1 | JMB-230 | 2015 |
| Thailand_Northeast | KX262987.1 | CHIKV/Homo sapiens/THA/SVO-451-96/1996 | 1996 |
| Thailand_Northeast | KX262988.1 | CHIKV/Homo sapiens/THA/6307-88/1988 | 1988 |
| Saint_Martin | KX262991.1 | CHIKV/Homo sapiens/SXM/H-20235-STMARTIN-2013/2003 | 2013 |
| Guadeloupe | KX262992.1 | CHIKV/Homo sapiens/GLP/YO-111213/2014 | 05/01/2014 |
| French_Guiana | KX262994.1 | CHIKV/Homo sapiens/GUF/YO-123223/2014 | 21/01/2014 |
| Colombia | KX496989.1 | Homo sapiens/COL/UF-1/2016 | 09/02/2016 |
| Haiti | KX702401.1 | Chikungunya virus Homo sapiens/Haiti-1/2014 | 02/06/2014 |
| Haiti | KX702402.1 | Chikungunya virus Homo sapiens/Haiti-2/2014 | 09/06/2014 |
| Dominican_Republic | KY272961.1 | N581 | 2014 |
| Dominican_Republic | KY272962.1 | N594 | 2014 |
| Dominican_Republic | KY272963.1 | N494 | 2014 |
| Dominican_Republic | KY272964.1 | N493 | 2014 |
| Dominican_Republic | KY272965.1 | N490 | 2014 |
| Dominican_Republic | KY272966.1 | N469 | 2014 |
| Dominican_Republic | KY272967.1 | N468 | 2014 |
| Dominican_Republic | KY272968.1 | N424 | 2014 |
| Dominican_Republic | KY272969.1 | N300 | 2014 |
| Dominican_Republic | KY272970.1 | N587 | 2014 |
| Haiti | KY415978.1 | Haiti-3/2014 | 29/05/2014 |
| Haiti | KY415979.1 | Haiti-4/2014 | 29/05/2014 |
| Haiti | KY415980.1 | Haiti-5/2014 | 05/06/2014 |
| Haiti | KY415981.1 | Haiti-6/2014 | 10/06/2014 |
| Haiti | KY415982.1 | Haiti-7/2014 | 11/06/2014 |
| Haiti | KY415983.1 | Haiti-8/2014 | 02/06/2014 |
| Haiti | KY415984.1 | Haiti-9/2014 | 24/06/2014 |
| Haiti | KY415985.1 | Haiti-10/2014 | 13/08/2014 |
| Trinidad_and_Tobago | KY435454.1 | 14.06638 | 11/2014 |
| Anguilla | KY435455.1 | 14.06523 | 12/11/2014 |
| Suriname | KY435456.1 | 14.06350 | 17/08/2014 |
| Montserrat | KY435457.1 | 14.06252 | 30/10/2014 |
| Guyana | KY435458.1 | 14.06121 | 03/11/2014 |
| Cayman_Islands | KY435459.1 | 14.05085 | 17/09/2014 |
| Cayman_Islands | KY435460.1 | 14.05081 | 06/07/2014 |
| Jamaica | KY435461.1 | 14.04561 | 24/08/2014 |
| Jamaica | KY435462.1 | 14.04558 | 25/08/2014 |
| Suriname | KY435463.1 | 14.04444 | 02/08/2014 |
| Barbados | KY435464.1 | 14.04425 | 15/08/2014 |
| Trinidad_and_Tobago | KY435465.1 | 14.04279 | 17/08/2014 |
| Barbados | KY435466.1 | 14.03985 | 06/08/2014 |
| Montserrat | KY435467.1 | 14.03844 | 24/07/2014 |
| Jamaica | KY435468.1 | 14.03837 | 06/08/2014 |
| Grenada | KY435469.1 | 14.03562 | 30/07/2014 |
| Bahamas | KY435470.1 | 14.02961 | 08/06/2014 |
| Turks_and_Caicos | KY435471.1 | 14.02585 | 11/06/2014 |
| Grenada | KY435472.1 | 14.02560 | 16/06/2014 |
| Saint_Lucia | KY435473.1 | 14.02557 | 22/05/2014 |
| Saint_Lucia | KY435474.1 | 14.02526 | 19/05/2014 |
| Saint_Vincent_and_the_Grenadines | KY435475.1 | 14.02346 | 22/05/2014 |
| Turks_and_Caicos | KY435476.1 | 14.02306 | 05/06/2014 |
| Guyana | KY435477.1 | 14.02217 | 31/05/2014 |
| Guyana | KY435478.1 | 14.02086 | 17/05/2014 |
| Antigua_and_Barbuda | KY435479.1 | 14.01526 | 28/04/2014 |
| Haiti | KY435480.1 | 14.01507 | 27/08/2014 |
| Saint_Lucia | KY435481.1 | 14.01349 | 22/04/2014 |
| Saint_Kitts_and_Nevis | KY435482.1 | 14.01152 | 11/03/2014 |
| Anguilla | KY435483.1 | 14.00686 | 12/02/2014 |
| Dominica | KY435484.1 | 14.00448 | 30/01/2014 |
| Dominica | KY435485.1 | 14.00324 | 28/01/2014 |
| British_Virgin_Islands | KY435486.1 | 14.00309 | 23/01/2014 |
| USA | KY575565.1 | CHIKV/Homo sapiens/USA/IDR1400024561/2014 | 2014 |
| USA | KY575566.1 | CHIKV/Homo sapiens/USA/IDR1400019200/2014 | 2014 |
| USA | KY575569.1 | CHIKV/Homo sapiens/USA/IDR1400021488/2014 | 2014 |
| USA | KY575572.1 | CHIKV/Homo sapiens/USA/IDR1400023906/2014 | 2014 |
| USA | KY575573.1 | CHIKV/Homo sapiens/USA/IDR1400021177/2014 | 2014 |
| USA_Broward | KY680347.1 | CHIKV/Homo sapiens/USA/CKVHL_47/2014 | 13/05/2014 |
| USA_Polk | KY680348.1 | CHIKV/Homo sapiens/USA/CKVHL_80/2014 | 05/09/2014 |
| USA_Orange | KY680349.1 | CHIKV/Homo sapiens/USA/CKVHL_97/2014 | 02/07/2014 |
| USA_Osceola | KY680350.1 | CHIKV/Homo sapiens/USA/CKVHL_61/2014 | 10/12/2014 |
| USA_Hillsborough | KY680351.1 | CHIKV/Homo sapiens/USA/CKVHL_26/2014 | 24/06/2014 |
| USA_Broward | KY680352.1 | CHIKV/Homo sapiens/USA/CKVHL_126/2014 | 15/05/2014 |
| USA_Seminole | KY680353.1 | CHIKV/Homo sapiens/USA/CKVHL_90/2014 | 25/08/2014 |
| USA_Hillsborough | KY680354.1 | CHIKV/Homo sapiens/USA/CKVHL_32/2014 | 08/05/2014 |
| USA_Palm_Beach | KY680355.1 | CHIKV/Homo sapiens/USA/CKVHL_125/2014 | 22/07/2014 |
| USA_Palm_Beach | KY680356.1 | CHIKV/Homo sapiens/USA/CKVHL_30/2014 | 21/07/2014 |
| USA_Orange | KY680357.1 | CHIKV/Homo sapiens/USA/CKVHL_51/2014 | 09/08/2014 |
| USA_Osceola | KY680358.1 | CHIKV/Homo sapiens/USA/CKVHL_33/2014 | 08/08/2014 |
| USA_Palm_Beach | KY680359.1 | CHIKV/Homo sapiens/USA/CKVHL_29/2014 | 04/05/2014 |
| USA_Broward | KY680360.1 | CHIKV/Homo sapiens/USA/CKVHL_114/2014 | 05/08/2014 |
| USA_Broward | KY680361.1 | CHIKV/Homo sapiens/USA/CKVHL_136/2014 | 26/05/2014 |
| USA_Dade | KY680362.1 | CHIKV/Homo sapiens/USA/CKVHL_81/2014 | 26/07/2014 |
| USA_Dade | KY680363.1 | CHIKV/Homo sapiens/USA/CKVHL_111/2014 | 15/06/2014 |
| USA_Dade | KY680364.1 | CHIKV/Homo sapiens/USA/CKVHL_139/2014 | 15/05/2014 |
| USA_Palm_Beach | KY680366.1 | CHIKV/Homo sapiens/USA/CKVHL_10/2015 | 24/07/2015 |
| USA_Dade | KY680367.1 | CHIKV/Homo sapiens/USA/CKVHL_49/2014 | 09/06/2014 |
| USA_Palm_Beach | KY680368.1 | CHIKV/Homo sapiens/USA/CKVHL_115/2014 | 01/12/2014 |
| USA_Dade | KY680369.1 | CHIKV/Homo sapiens/USA/CKVHL_22/2014 | 24/05/2014 |
| USA_Orange | KY680370.1 | CHIKV/Homo sapiens/USA/CKVHL_57/2014 | 03/10/2014 |
| USA_Palm_Beach | KY680371.1 | CHIKV/Homo sapiens/USA/CKVHL_95/2014 | 20/08/2014 |
| USA_Broward | KY680372.1 | CHIKV/Homo sapiens/USA/CKVHL_86/2014 | 04/06/2014 |
| USA_Duval | KY680373.1 | CHIKV/Homo sapiens/USA/CKVHL_113/2014 | 22/10/2014 |
| USA_Osceola | KY680374.1 | CHIKV/Homo sapiens/USA/CKVHL_89/2014 | 06/12/2014 |
| USA_Osceola | KY680375.1 | CHIKV/Homo sapiens/USA/CKVHL_24/2014 | 04/06/2014 |
| USA_Polk | KY680376.1 | CHIKV/Homo sapiens/USA/CKVHL_85/2014 | 08/10/2014 |
| USA_Polk | KY680377.1 | CHIKV/Homo sapiens/USA/CKVHL_28/2014 | 16/08/2014 |
| USA_Collier | KY680378.1 | CHIKV/Homo sapiens/USA/CKVHL_103/2014 | 19/10/2014 |
| USA_Hillsborough | KY680379.1 | CHIKV/Homo sapiens/USA/CKVHL_53/2014 | 16/09/2014 |
| USA | KY680380.1 | CHIKV/Homo sapiens/USA/CKVHL_104/2014 | 13/10/2014 |
| USA_Osceola | KY680381.1 | CHIKV/Homo sapiens/USA/CKVHL_79/2014 | 21/10/2014 |
| USA_Duval | KY680382.1 | CHIKV/Homo sapiens/USA/CKVHL_135/2014 | 04/06/2014 |
| USA_Palm_Beach | KY680383.1 | CHIKV/Homo sapiens/USA/CKVHL_121/2014 | 20/08/2014 |
| USA_Orange | KY680384.1 | CHIKV/Homo sapiens/USA/CKVHL_03/2014 | 25/06/2014 |
| USA_Polk | KY680385.1 | CHIKV/Homo sapiens/USA/CKVHL_70/2014 | 25/11/2014 |
| USA_Broward | KY680386.1 | CHIKV/Homo sapiens/USA/CKVHL_16/2014 | 24/09/2014 |
| USA_Osceola | KY680387.1 | CHIKV/Homo sapiens/USA/CKVHL_18/2014 | 14/10/2014 |
| USA_Polk | KY680388.1 | CHIKV/Homo sapiens/USA/CKVHL_76/2014 | 02/10/2014 |
| USA_Osceola | KY680389.1 | CHIKV/Homo sapiens/USA/CKVHL_59/2015 | 14/07/2015 |
| USA_Hillsborough | KY680390.1 | CHIKV/Homo sapiens/USA/CKVHL_23/2014 | 07/05/2014 |
| USA_Orange | KY680391.1 | CHIKV/Homo sapiens/USA/CKVHL_72/2014 | 24/10/2014 |
| USA_Osceola | KY680392.1 | CHIKV/Homo sapiens/USA/CKVHL_09/2014 | 19/10/2014 |
| USA_Volusia | KY680393.1 | CHIKV/Homo sapiens/USA/CKVHL_42/2014 | 17/09/2014 |
| USA_Osceola | KY680394.1 | CHIKV/Homo sapiens/USA/CKVHL_99/2014 | 05/10/2014 |
| USA_Dade | KY680395.1 | CHIKV/Homo sapiens/USA/CKVHL_08/2014 | 21/07/2014 |
| USA_Osceola | KY680396.1 | CHIKV/Homo sapiens/USA/CKVHL_35/2014 | 02/09/2014 |
| USA_Dade | KY680397.1 | CHIKV/Homo sapiens/USA/CKVHL_01/2014 | 23/05/2014 |
| USA_Palm_Beach | KY680398.1 | CHIKV/Homo sapiens/USA/CKVHL_119/2014 | 05/08/2014 |
| USA_Duval | KY680399.1 | CHIKV/Homo sapiens/USA/CKVHL_120/2014 | 05/09/2014 |
| USA_Pasco | KY680400.1 | CHIKV/Homo sapiens/USA/CKVHL_27/2014 | 15/07/2014 |
| USA | KY680401.1 | CHIKV/Homo sapiens/USA/CKVHL_101/2014 | 22/09/2014 |
| USA_Palm_Beach | KY680402.1 | CHIKV/Homo sapiens/USA/CKVHL_82/2014 | 27/07/2014 |
| USA_Osceola | KY680403.1 | CHIKV/Homo sapiens/USA/CKVHL_91/2014 | 14/06/2014 |
| USA_Orange | KY680404.1 | CHIKV/Homo sapiens/USA/CKVHL_67/2014 | 17/09/2014 |
| USA_Osceola | KY680405.1 | CHIKV/Homo sapiens/USA/CKVHL_96/2014 | 18/06/2014 |
| USA_Polk | KY680406.1 | CHIKV/Homo sapiens/USA/CKVHL_05/2014 | 08/08/2014 |
| USA_Duval | KY680407.1 | CHIKV/Homo sapiens/USA/CKVHL_116/2014 | 20/11/2014 |
| USA_Dade | KY680408.1 | CHIKV/Homo sapiens/USA/CKVHL_34/2014 | 26/06/2014 |
| USA_Duval | KY680409.1 | CHIKV/Homo sapiens/USA/CKVHL_124/2014 | 21/10/2014 |
| USA_Osceola | KY680410.1 | CHIKV/Homo sapiens/USA/CKVHL_20/2014 | 22/08/2014 |
| USA_Orange | KY680411.1 | CHIKV/Homo sapiens/USA/CKVHL_94/2014 | 04/09/2014 |
| USA_Dade | KY680412.1 | CHIKV/Homo sapiens/USA/CKVHL_88/2014 | 28/08/2014 |
| USA_Duval | KY680413.1 | CHIKV/Homo sapiens/USA/CKVHL_128/2014 | 17/09/2014 |
| USA_Seminole | KY680414.1 | CHIKV/Homo sapiens/USA/CKVHL_21/2014 | 13/09/2014 |
| Nicaragua_Managua | KY703888.1 | CHIKV/Homo sapiens/NIC/1885.1D/2015 | 15/08/2015 |
| Nicaragua_Managua | KY703889.1 | CHIKV/Homo sapiens/NIC/1863.1C/2015 | 05/08/2015 |
| Nicaragua_Masaya | KY703890.1 | CHIKV/Homo sapiens/NIC/11132-15/2015 | 23/09/2015 |
| Nicaragua_Rivas | KY703891.1 | CHIKV/Homo sapiens/NIC/11006-15/2015 | 17/09/2015 |
| Nicaragua_Managua | KY703892.1 | CHIKV/Homo sapiens/NIC/1988.1C/2015 | 26/11/2015 |
| Nicaragua_Managua | KY703893.1 | CHIKV/Homo sapiens/NIC/1908.1C/2015 | 02/09/2015 |
| Nicaragua_Managua | KY703894.1 | CHIKV/Homo sapiens/NIC/6446.1LA1/2015 | 09/10/2015 |
| Nicaragua_Managua | KY703895.1 | CHIKV/Homo sapiens/NIC/1346.12.A.1/2015 | 26/11/2015 |
| Nicaragua_Managua | KY703896.1 | CHIKV/Homo sapiens/NIC/1816.1E/2014 | 18/12/2014 |
| Nicaragua_Managua | KY703897.1 | CHIKV/Homo sapiens/NIC/1825.1C/2015 | 16/01/2015 |
| Nicaragua_Nuevo_Segovia | KY703899.1 | CHIKV/Homo sapiens/NIC/12438-15/2015 | 20/10/2015 |
| Nicaragua_Managua | KY703900.1 | CHIKV/Homo sapiens/NIC/1993.1C/2015 | 14/12/2015 |
| Nicaragua_Managua | KY703901.1 | CHIKV/Homo sapiens/NIC/1838.1C/2015 | 26/01/2015 |
| Nicaragua_Managua | KY703902.1 | CHIKV/Homo sapiens/NIC/CKVGH11/2015 | 05/09/2015 |
| Nicaragua_Managua | KY703903.1 | CHIKV/Homo sapiens/NIC/1912.1C/2015 | 05/09/2015 |
| Nicaragua_Managua | KY703904.1 | CHIKV/Homo sapiens/NIC/5823.11A1/2014 | 04/10/2014 |
| Nicaragua_Managua | KY703905.1 | CHIKV/Homo sapiens/NIC/1920.1C/2015 | 17/09/2015 |
| Nicaragua_Managua | KY703906.1 | CHIKV/Homo sapiens/NIC/1914.1C/2015 | 08/09/2015 |
| Nicaragua_Managua | KY703907.1 | CHIKV/Homo sapiens/NIC/996.12.A.1/2015 | 15/07/2015 |
| Nicaragua_Managua | KY703908.1 | CHIKV/Homo sapiens/NIC/6826.11A1/2014 | 02/12/2014 |
| Nicaragua_Managua | KY703909.1 | CHIKV/Homo sapiens/NIC/1842.1C/2015 | 28/01/2015 |
| Nicaragua_Nuevo_Segovia | KY703910.1 | CHIKV/Homo sapiens/NIC/12062-15/2015 | 18/10/2015 |
| Nicaragua_Managua | KY703911.1 | CHIKV/Homo sapiens/NIC/1167.12.A.1/2015 | 17/12/2015 |
| Nicaragua_Managua | KY703913.1 | CHIKV/Homo sapiens/NIC/1878.1C/2015 | 13/08/2015 |
| Nicaragua_Managua | KY703914.1 | CHIKV/Homo sapiens/NIC/1886.1D/2015 | 15/08/2015 |
| Nicaragua_Managua | KY703915.1 | CHIKV/Homo sapiens/NIC/1857.1C/2015 | 03/08/2015 |
| Nicaragua_Managua | KY703916.1 | CHIKV/Homo sapiens/NIC/6556.12.A.1/2015 | 04/08/2015 |
| Nicaragua_Esteli | KY703917.1 | CHIKV/Homo sapiens/NIC/14-16/2015 | 08/01/2016 |
| Nicaragua_Managua | KY703918.1 | CHIKV/Homo sapiens/NIC/1839.1C/2015 | 26/01/2015 |
| Nicaragua_Managua | KY703919.1 | CHIKV/Homo sapiens/NIC/1844.1C/2015 | 29/01/2015 |
| Nicaragua_Granada | KY703920.1 | CHIKV/Homo sapiens/NIC/13491-15/2015 | 16/11/2015 |
| Nicaragua_Managua | KY703921.1 | CHIKV/Homo sapiens/NIC/1967.1C/2015 | 04/11/2015 |
| Nicaragua_Managua | KY703922.1 | CHIKV/Homo sapiens/NIC/7074.12A1/2015 | 06/09/2015 |
| Nicaragua_Managua | KY703923.1 | CHIKV/Homo sapiens/NIC/1925.1C/2015 | 19/09/2015 |
| Nicaragua_Managua | KY703924.1 | CHIKV/Homo sapiens/NIC/1879.1C/2015 | 13/08/2015 |
| Nicaragua_Managua | KY703925.1 | CHIKV/Homo sapiens/NIC/5201.12A1/2015 | 06/09/2015 |
| Nicaragua_Managua | KY703926.1 | CHIKV/Homo sapiens/NIC/1891.1C/2015 | 20/08/2015 |
| Nicaragua_Managua | KY703927.1 | CHIKV/Homo sapiens/NIC/1822.1C/2015 | 15/01/2015 |
| Nicaragua_Managua | KY703928.1 | CHIKV/Homo sapiens/NIC/1847.1C/2015 | 30/01/2015 |
| Nicaragua_Managua | KY703930.1 | CHIKV/Homo sapiens/NIC/4912.12.A.1/2015 | 24/11/2015 |
| Nicaragua_Managua | KY703931.1 | CHIKV/Homo sapiens/NIC/1924.1C/2015 | 19/09/2015 |
| Nicaragua_Managua | KY703932.1 | CHIKV/Homo sapiens/NIC/1890.1D/2015 | 19/08/2015 |
| Nicaragua_Managua | KY703933.1 | CHIKV/Homo sapiens/NIC/8476.1LA1/2015 | 09/10/2015 |
| Nicaragua_Managua | KY703934.1 | CHIKV/Homo sapiens/NIC/8065.1LA1/2015 | 27/10/2015 |
| Nicaragua_Managua | KY703935.1 | CHIKV/Homo sapiens/NIC/4900.12.A.1/2015 | 02/12/2015 |
| Nicaragua_Managua | KY703936.1 | CHIKV/Homo sapiens/NIC/1497.1LA1/2015 | 07/10/2015 |
| Nicaragua_Managua | KY703937.1 | CHIKV/Homo sapiens/NIC/1956.1C/2015 | 15/10/2015 |
| Nicaragua_Managua | KY703938.1 | CHIKV/Homo sapiens/NIC/1852.1D/2015 | 18/02/2015 |
| Nicaragua_Managua | KY703940.1 | CHIKV/Homo sapiens/NIC/1756.1C/2014 | 28/10/2014 |
| Nicaragua_Managua | KY703942.1 | CHIKV/Homo sapiens/NIC/86.12.A.1/2015 | 04/08/2015 |
| Nicaragua_Managua | KY703943.1 | CHIKV/Homo sapiens/NIC/1948.1C/2015 | 10/10/2015 |
| Nicaragua_Managua | KY703944.1 | CHIKV/Homo sapiens/NIC/1938.1C/2015 | 03/10/2015 |
| Nicaragua_Managua | KY703945.1 | CHIKV/Homo sapiens/NIC/1937.1C/2015 | 02/10/2015 |
| Nicaragua_Managua | KY703946.1 | CHIKV/Homo sapiens/NIC/1952.1C/2015 | 12/10/2015 |
| Nicaragua_Managua | KY703947.1 | CHIKV/Homo sapiens/NIC/1778.1C/2014 | 19/11/2014 |
| Nicaragua_Managua | KY703948.1 | CHIKV/Homo sapiens/NIC/1889.1D/2015 | 18/08/2015 |
| Nicaragua_Managua | KY703949.1 | CHIKV/Homo sapiens/NIC/1824.1C/2015 | 15/01/2015 |
| Nicaragua_Managua | KY703950.1 | CHIKV/Homo sapiens/NIC/1790.1C/2014 | 25/11/2014 |
| Nicaragua_Managua | KY703952.1 | CHIKV/Homo sapiens/NIC/CKVGH13/2015 | 03/09/2015 |
| Nicaragua_Managua | KY703954.1 | CHIKV/Homo sapiens/NIC/1758.1C/2014 | 05/11/2014 |
| Nicaragua_Managua | KY703955.1 | CHIKV/Homo sapiens/NIC/2741.12.A.1/2015 | 17/08/2015 |
| Nicaragua_Managua | KY703956.1 | CHIKV/Homo sapiens/NIC/7172.11A1/2014 | 28/10/2014 |
| Nicaragua_Managua | KY703957.1 | CHIKV/Homo sapiens/NIC/7555.12A1/2015 | 05/09/2015 |
| Nicaragua_Managua | KY703958.1 | CHIKV/Homo sapiens/NIC/1985.1C/2015 | 25/11/2015 |
| Nicaragua_Managua | KY703959.1 | CHIKV/Homo sapiens/NIC/1800.1D/2014 | 03/12/2014 |
| Nicaragua_Managua | KY703960.1 | CHIKV/Homo sapiens/NIC/1864.1C/2015 | 05/08/2015 |
| Nicaragua_Managua | KY703961.1 | CHIKV/Homo sapiens/NIC/7101.12.A./2015 | 06/08/2015 |
| Nicaragua_Nuevo_Segovia | KY703962.1 | CHIKV/Homo sapiens/NIC/11630-15/2015 | 01/10/2015 |
| Nicaragua_Managua | KY703963.1 | CHIKV/Homo sapiens/NIC/1913.1C/2015 | 07/09/2015 |
| Nicaragua_Managua | KY703965.1 | CHIKV/Homo sapiens/NIC/1909.1C/2015 | 03/09/2015 |
| Nicaragua_Managua | KY703966.1 | CHIKV/Homo sapiens/NIC/4036.12.A.1/2015 | 10/12/2015 |
| Nicaragua_Managua | KY703967.1 | CHIKV/Homo sapiens/NIC/8976.12.A.1/2015 | 12/08/2015 |
| Nicaragua_Managua | KY703968.1 | CHIKV/Homo sapiens/NIC/1837.1D/2015 | 23/01/2015 |
| Nicaragua_Managua | KY703969.1 | CHIKV/Homo sapiens/NIC/1773.1C/2014 | 14/11/2014 |
| Nicaragua_Carazo | KY703970.1 | CHIKV/Homo sapiens/NIC/14905-15/2015 | 28/11/2015 |
| Nicaragua_Carazo | KY703971.1 | CHIKV/Homo sapiens/NIC/14906-15/2015 | 28/11/2015 |
| Nicaragua_Managua | KY703972.1 | CHIKV/Homo sapiens/NIC/1805.1E/2014 | 09/12/2014 |
| Nicaragua_Managua | KY703973.1 | CHIKV/Homo sapiens/NIC/6523.12.A.1/2015 | 06/11/2015 |
| Nicaragua_Managua | KY703974.1 | CHIKV/Homo sapiens/NIC/1910.1C/2015 | 03/09/2015 |
| Nicaragua_Masaya | KY703975.1 | CHIKV/Homo sapiens/NIC/11091-15/2015 | 22/09/2015 |
| Nicaragua_Managua | KY703976.1 | CHIKV/Homo sapiens/NIC/6450.12A1/2015 | 05/09/2015 |
| Nicaragua_Managua | KY703977.1 | CHIKV/Homo sapiens/NIC/1835.12A1/2015 | 03/08/2015 |
| Nicaragua_Masaya | KY703978.1 | CHIKV/Homo sapiens/NIC/14648-15/2015 | 02/12/2015 |
| Nicaragua_Matagalpa | KY703979.1 | CHIKV/Homo sapiens/NIC/11093-15/2015 | 22/09/2015 |
| Nicaragua_Managua | KY703980.1 | CHIKV/Homo sapiens/NIC/6638.12A1/2015 | 06/09/2015 |
| Nicaragua_Managua | KY703982.1 | CHIKV/Homo sapiens/NIC/4040.12.A.1/2015 | 30/11/2015 |
| Nicaragua_Managua | KY703983.1 | CHIKV/Homo sapiens/NIC/1809.1E/2014 | 11/12/2014 |
| Nicaragua_Managua | KY703984.1 | CHIKV/Homo sapiens/NIC/1794.1C/2014 | 27/11/2014 |
| Nicaragua_Managua | KY703985.1 | CHIKV/Homo sapiens/NIC/1880.1D/2015 | 13/08/2015 |
| Nicaragua_Managua | KY703986.1 | CHIKV/Homo sapiens/NIC/1918.1C/2015 | 16/09/2015 |
| Nicaragua_Managua | KY703987.1 | CHIKV/Homo sapiens/NIC/1367.12.A.1/2015 | 04/11/2015 |
| Nicaragua_Managua | KY703988.1 | CHIKV/Homo sapiens/NIC/1793.1D/2014 | 27/11/2014 |
| Nicaragua_Managua | KY703989.1 | CHIKV/Homo sapiens/NIC/1802.1D/2014 | 03/12/2014 |
| Nicaragua_Managua | KY703990.1 | CHIKV/Homo sapiens/NIC/1983.1C/2015 | 21/11/2015 |
| Nicaragua_Managua | KY703991.1 | CHIKV/Homo sapiens/NIC/24044.1LA1/2015 | 09/10/2015 |
| Nicaragua_Managua | KY703992.1 | CHIKV/Homo sapiens/NIC/5695.12A1/2015 | 05/08/2015 |
| Nicaragua_Managua | KY703993.1 | CHIKV/Homo sapiens/NIC/1760.1C/2014 | 07/11/2014 |
| Nicaragua_Esteli | KY703994.1 | CHIKV/Homo sapiens/NIC/13724-15/2015 | 18/11/2015 |
| Nicaragua_Managua | KY703995.1 | CHIKV/Homo sapiens/NIC/1882.1D/2015 | 13/08/2015 |
| Nicaragua_Managua | KY703996.1 | CHIKV/Homo sapiens/NIC/5024.12.A.1/2015 | 26/12/2015 |
| Nicaragua_Managua | KY703997.1 | CHIKV/Homo sapiens/NIC/4354.12.A.1/2015 | 19/12/2015 |
| Nicaragua_Managua | KY703998.1 | CHIKV/Homo sapiens/NIC/7156.12A1/2015 | 06/08/2015 |
| Nicaragua_Managua | KY703999.1 | CHIKV/Homo sapiens/NIC/1828.1C/2015 | 19/01/2015 |
| Nicaragua_Managua | KY704000.1 | CHIKV/Homo sapiens/NIC/1862.1C/2015 | 05/08/2015 |
| Nicaragua_Managua | KY704001.1 | CHIKV/Homo sapiens/NIC/1829.1C/2015 | 21/01/2015 |
| Nicaragua_Managua | KY704002.1 | CHIKV/Homo sapiens/NIC/1823.1C/2015 | 15/01/2015 |
| Singapore | KY883764.1 | SGEHICH02971Y13 | 01/2013 |
| Thailand | LC259082.1 | BaH306-NIID | 1958 |
| Indonesia | LC259083.1 | CHIKV/Hu/Indonesia/NIID58/2009 | 11/09/2009 |
| Philippines | LC259084.1 | CHIKV/Hu/Philippines/NIID165/2012 | 25/09/2012 |
| Indonesia | LC259085.1 | CHIKV/Hu/Indonesia/NIID181/2012 | 15/10/2012 |
| Indonesia | LC259086.1 | CHIKV/Hu/Indonesia/NIID108/2013 | 01/07/2013 |
| Indonesia | LC259087.1 | CHIKV/Hu/Indonesia/NIID112/2013 | 06/07/2013 |
| Tonga | LC259088.1 | CHIKV/Hu/Tonga/NIID41/2014 | 25/03/2014 |
| Dominica | LC259089.1 | CHIKV/Hu/Commonwealth of Dominica/NIID73/2014 | 23/06/2014 |
| Colombia | LC259090.1 | CHIKV/Hu/Colombia/NIID02/2015 | 06/01/2015 |
| Indonesia | LC259091.1 | CHIKV/Hu/Indonesia/NIID35/2015 | 07/05/2015 |
| Cuba | LC259092.1 | CHIKV/Hu/Cuba/NIID25/2016 | 28/02/2016 |
| Martinique | LN898093.1 | M100 | 12/2013 |
| Guadeloupe | LN898094.1 | G100 | 01/2014 |
| Martinique | LN898095.1 | M101 | 01/2014 |
| Martinique | LN898096.1 | M102 | 01/2014 |
| Martinique | LN898097.1 | G101 | 01/2014 |
| Guadeloupe | LN898098.1 | G102 | 01/2014 |
| Guadeloupe | LN898099.1 | G103 | 01/2014 |
| Martinique | LN898100.1 | M103 | 01/2014 |
| Martinique | LN898101.1 | M104 | 01/2014 |
| Guadeloupe | LN898102.1 | G104 | 01/2014 |
| Guadeloupe | LN898103.1 | G105 | 01/2014 |
| Martinique | LN898104.1 | M105 | 01/2014 |
| Martinique | LN898105.1 | M106 | 01/2014 |
| Martinique | LN898106.1 | M107 | 01/2014 |
| Martinique | LN898107.1 | M108 | 01/2014 |
| Martinique | LN898108.1 | M109 | 01/2014 |
| Martinique | LN898109.1 | M110 | 01/2014 |
| Guadeloupe | LN898110.1 | G106 | 01/2014 |
| Guadeloupe | LN898111.1 | G107 | 01/2014 |
| Martinique | LN898112.1 | M111 | 01/2014 |
| Puerto_Rico | MF001505.1 | JFRO_01 | 2015 |
| Puerto_Rico | MF001506.1 | JFRO_02 | 2015 |
| Puerto_Rico | MF001507.1 | JFRO_03 | 2015 |
| Puerto_Rico | MF001508.1 | JFRO_04 | 2015 |
| Puerto_Rico | MF001509.1 | JFRO_05 | 2015 |
| Puerto_Rico | MF001510.1 | JFRO_06 | 2015 |
| Puerto_Rico | MF001511.1 | JFRO_07 | 2015 |
| Puerto_Rico | MF001512.1 | JFRO_08 | 2015 |
| Puerto_Rico | MF001513.1 | JFRO_09 | 2015 |
| Puerto_Rico | MF001514.1 | JFRO_10 | 2015 |
| Puerto_Rico | MF001515.1 | JFRO_11 | 2015 |
| Puerto_Rico | MF001516.1 | JFRO_12 | 2015 |
| Puerto_Rico | MF001517.1 | JFRO_13 | 2015 |
| Puerto_Rico | MF001518.1 | JFRO_14 | 2015 |
| Puerto_Rico | MF001519.1 | JFRO_15 | 2015 |
| Samoa | MF773559.1 | Samoa 2014 | 2014 |
| Caribbean | MF773560.1 | Caribbean 2014 | 2014 |
| Indonesia_Bali | MF773561.1 | Bali 2011 | 2011 |
| Kiribati | MF773562.1 | Kiribati 2015 | 2015 |
| Philippines | MF773563.1 | Philippines 2014 | 2014 |
| Philippines | MF773564.1 | Philippines 2016 | 2016 |
| Timor/Leste | MF773565.1 | ET2010 | 2010 |
| St_Martin | MG208125.1 | H20235/St. Martin/2013 | 2013 |
| China | MG664851.1 | SZ1239 | 2012 |
| Mexico_Reynosa | MG822707.1 | CH-R-1972 | 2015 |
| Mexico_Reynosa | MG822708.1 | CH-R-2295 | 2015 |
| Mexico_Reynosa | MG921596.1 | CH-R-1950 | 2015 |
| Haiti | MG967666.1 | Homo sapiens/Haiti-11/2014 | 06/2014 |
| China | MH670649.1 | Chikungunya virus complete genome | 19/11/2009 |
