# Supplementary material for: Genetic Variability of Chikungunya Virus in Southern Mexico
Source: Viruses. 2019 Aug 5;11(8):714. doi: 10.3390/v11080714 (PMC6722872; doi:10.3390/v11080714)
Supplement: Supplementary file 1 [file viruses-11-00714-s001.zip › Suplementary/Supplementary Table 3.docx]

**Table S3.** Negative selected sites in the studied CHIKV Asian lineages ^a^.

| **Dataset** | **Method** | **Number of selected sites** | **Codon position ^d^** | **Significance** |
| --- | --- | --- | --- | --- |
| Asian | SLAC | 29 | 743 | 0.0005 ^b^ |
|  |  |  | 73 | 0.0024 ^b^ |
|  |  |  | 8 | 0.0041 ^b^ |
|  |  |  | 712 | 0.0041 ^b^ |
|  |  |  | 796 | 0.0057 ^b^ |
|  |  |  | 131 | 0.0067 ^b^ |
|  |  |  | 552 | 0.0070 ^b^ |
|  |  |  | 365 | 0.0123 ^b^ |
|  |  |  | 324 | 0.0189 ^b^ |
|  |  |  | 920 | 0.0209 ^b^ |
|  |  |  | 62 | 0.0209 ^b^ |
|  |  |  | 865 | 0.0211 ^b^ |
|  |  |  | 333 | 0.0212 ^b^ |
|  |  |  | 700 | 0.0222 ^b^ |
|  |  |  | 214 | 0.0223 ^b^ |
|  |  |  | 203 | 0.0243 ^b^ |
|  |  |  | 69 | 0.0253 ^b^ |
|  |  |  | 141 | 0.0267 ^b^ |
|  |  |  | 694 | 0.0268 ^b^ |
|  |  |  | 99 | 0.0268 ^b^ |
|  |  |  | 26 | 0.0270 ^b^ |
|  |  |  | 776 | 0.0270 ^b^ |
|  |  |  | 606 | 0.0288 ^b^ |
|  |  |  | 911 | 0.0334 ^b^ |
|  |  |  | 155 | 0.0370 ^b^ |
|  |  |  | 244 | 0.0370 ^b^ |
|  |  |  | 429 | 0.0370 ^b^ |
|  |  |  | 628 | 0.0370 ^b^ |
|  |  |  | 905 | 0.0370 ^b^ |
|  | FEL | 80 | 712 | < 0.001 ^b^ |
|  |  |  | 8 | 0.001 ^b^ |
|  |  |  | 73 | 0.001 ^b^ |
|  |  |  | 743 | 0.001 ^b^ |
|  |  |  | 796 | 0.002 ^b^ |
|  |  |  | 141 | 0.003 ^b^ |
|  |  |  | 155 | 0.003 ^b^ |
|  |  |  | 131 | 0.004 ^b^ |
|  |  |  | 628 | 0.004 ^b^ |
|  |  |  | 552 | 0.005 ^b^ |
|  |  |  | 911 | 0.006 ^b^ |
|  |  |  | 324 | 0.007 ^b^ |
|  |  |  | 905 | 0.007 ^b^ |
|  |  |  | 26 | 0.008 ^b^ |
|  |  |  | 99 | 0.008 ^b^ |
|  |  |  | 920 | 0.008 ^b^ |
|  |  |  | 365 | 0.009 ^b^ |
|  |  |  | 694 | 0.01 ^b^ |
|  |  |  | 606 | 0.011 ^b^ |
|  |  |  | 243 | 0.012 ^b^ |
|  |  |  | 429 | 0.012 ^b^ |
|  |  |  | 469 | 0.013 ^b^ |
|  |  |  | 506 | 0.013 ^b^ |
|  |  |  | 203 | 0.015 ^b^ |
|  |  |  | 75 | 0.016 ^b^ |
|  |  |  | 100 | 0.016 ^b^ |
|  |  |  | 410 | 0.016 ^b^ |
|  |  |  | 571 | 0.016 ^b^ |
|  |  |  | 676 | 0.016 ^b^ |
|  |  |  | 776 | 0.016 ^b^ |
|  |  |  | 62 | 0.017 ^b^ |
|  |  |  | 244 | 0.017 ^b^ |
|  |  |  | 865 | 0.017 ^b^ |
|  |  |  | 794 | 0.018 ^b^ |
|  |  |  | 41 | 0.019 ^b^ |
|  |  |  | 355 | 0.02 ^b^ |
|  |  |  | 600 | 0.02 ^b^ |
|  |  |  | 12 | 0.021 ^b^ |
|  |  |  | 69 | 0.022 ^b^ |
|  |  |  | 364 | 0.022 ^b^ |
|  |  |  | 655 | 0.022 ^b^ |
|  |  |  | 849 | 0.022 ^b^ |
|  |  |  | 700 | 0.023 ^b^ |
|  |  |  | 80 | 0.024 ^b^ |
|  |  |  | 563 | 0.024 ^b^ |
|  |  |  | 793 | 0.024 ^b^ |
|  |  |  | 333 | 0.025 ^b^ |
|  |  |  | 28 | 0.027 ^b^ |
|  |  |  | 214 | 0.027 ^b^ |
|  |  |  | 316 | 0.027 ^b^ |
|  |  |  | 523 | 0.027 ^b^ |
|  |  |  | 546 | 0.027 ^b^ |
|  |  |  | 917 | 0.027 ^b^ |
|  |  |  | 115 | 0.028 ^b^ |
|  |  |  | 741 | 0.029 ^b^ |
|  |  |  | 192 | 0.03 ^b^ |
|  |  |  | 396 | 0.03 ^b^ |
|  |  |  | 454 | 0.03 ^b^ |
|  |  |  | 872 | 0.03 ^b^ |
|  |  |  | 114 | 0.031 ^b^ |
|  |  |  | 180 | 0.032 ^b^ |
|  |  |  | 201 | 0.032 ^b^ |
|  |  |  | 537 | 0.032 ^b^ |
|  |  |  | 566 | 0.032 ^b^ |
|  |  |  | 791 | 0.032 ^b^ |
|  |  |  | 880 | 0.032 ^b^ |
|  |  |  | 669 | 0.033 ^b^ |
|  |  |  | 175 | 0.034 ^b^ |
|  |  |  | 508 | 0.034 ^b^ |
|  |  |  | 424 | 0.035 ^b^ |
|  |  |  | 815 | 0.035 ^b^ |
|  |  |  | 756 | 0.036 ^b^ |
|  |  |  | 521 | 0.038 ^b^ |
|  |  |  | 731 | 0.04 ^b^ |
|  |  |  | 170 | 0.045 ^b^ |
|  |  |  | 453 | 0.048 ^b^ |
|  |  |  | 43 | 0.05 ^b^ |
|  |  |  | 71 | 0.05 ^b^ |
|  |  |  | 635 | 0.05 ^b^ |
|  |  |  | 658 | 0.05 ^b^ |
|  | FUBAR | 120 | 8 | 1 ^c^ |
|  |  |  | 73 | 1 ^c^ |
|  |  |  | 712 | 1 ^c^ |
|  |  |  | 743 | 1 ^c^ |
|  |  |  | 131 | 0.999 ^c^ |
|  |  |  | 796 | 0.999 ^c^ |
|  |  |  | 155 | 0.998 ^c^ |
|  |  |  | 365 | 0.998 ^c^ |
|  |  |  | 469 | 0.998 ^c^ |
|  |  |  | 552 | 0.998 ^c^ |
|  |  |  | 628 | 0.998 ^c^ |
|  |  |  | 324 | 0.997 ^c^ |
|  |  |  | 141 | 0.997 ^c^ |
|  |  |  | 911 | 0.997 ^c^ |
|  |  |  | 26 | 0.996 ^c^ |
|  |  |  | 99 | 0.996 ^c^ |
|  |  |  | 429 | 0.996 ^c^ |
|  |  |  | 694 | 0.996 ^c^ |
|  |  |  | 905 | 0.996 ^c^ |
|  |  |  | 920 | 0.996 ^c^ |
|  |  |  | 606 | 0.995 ^c^ |
|  |  |  | 244 | 0.995 ^c^ |
|  |  |  | 776 | 0.995 ^c^ |
|  |  |  | 731 | 0.994 ^c^ |
|  |  |  | 203 | 0.994 ^c^ |
|  |  |  | 62 | 0.994 ^c^ |
|  |  |  | 865 | 0.994 ^c^ |
|  |  |  | 69 | 0.993 ^c^ |
|  |  |  | 214 | 0.993 ^c^ |
|  |  |  | 333 | 0.993 ^c^ |
|  |  |  | 700 | 0.993 ^c^ |
|  |  |  | 180 | 0.992 ^c^ |
|  |  |  | 912 | 0.99 ^c^ |
|  |  |  | 243 | 0.99 ^c^ |
|  |  |  | 506 | 0.99 ^c^ |
|  |  |  | 75 | 0.989 ^c^ |
|  |  |  | 794 | 0.989 ^c^ |
|  |  |  | 12 | 0.988 ^c^ |
|  |  |  | 41 | 0.988 ^c^ |
|  |  |  | 600 | 0.988 ^c^ |
|  |  |  | 655 | 0.988 ^c^ |
|  |  |  | 80 | 0.987 ^c^ |
|  |  |  | 100 | 0.987 ^c^ |
|  |  |  | 364 | 0.987 ^c^ |
|  |  |  | 410 | 0.987 ^c^ |
|  |  |  | 563 | 0.987 ^c^ |
|  |  |  | 571 | 0.987 ^c^ |
|  |  |  | 676 | 0.987 ^c^ |
|  |  |  | 114 | 0.986 ^c^ |
|  |  |  | 316 | 0.986 ^c^ |
|  |  |  | 523 | 0.986 ^c^ |
|  |  |  | 793 | 0.985 ^c^ |
|  |  |  | 115 | 0.984 ^c^ |
|  |  |  | 28 | 0.984 ^c^ |
|  |  |  | 546 | 0.984 ^c^ |
|  |  |  | 849 | 0.984 ^c^ |
|  |  |  | 917 | 0.984 ^c^ |
|  |  |  | 586 | 0.983 ^c^ |
|  |  |  | 192 | 0.983 ^c^ |
|  |  |  | 396 | 0.983 ^c^ |
|  |  |  | 424 | 0.983 ^c^ |
|  |  |  | 454 | 0.983 ^c^ |
|  |  |  | 537 | 0.983 ^c^ |
|  |  |  | 566 | 0.983 ^c^ |
|  |  |  | 791 | 0.983 ^c^ |
|  |  |  | 872 | 0.983 ^c^ |
|  |  |  | 880 | 0.983 ^c^ |
|  |  |  | 175 | 0.982 ^c^ |
|  |  |  | 201 | 0.982 ^c^ |
|  |  |  | 508 | 0.982 ^c^ |
|  |  |  | 669 | 0.982 ^c^ |
|  |  |  | 756 | 0.982 ^c^ |
|  |  |  | 815 | 0.982 ^c^ |
|  |  |  | 521 | 0.981 ^c^ |
|  |  |  | 741 | 0.981 ^c^ |
|  |  |  | 43 | 0.979 ^c^ |
|  |  |  | 71 | 0.979 ^c^ |
|  |  |  | 104 | 0.979 ^c^ |
|  |  |  | 117 | 0.979 ^c^ |
|  |  |  | 635 | 0.979 ^c^ |
|  |  |  | 658 | 0.979 ^c^ |
|  |  |  | 170 | 0.978 ^c^ |
|  |  |  | 779 | 0.977 ^c^ |
|  |  |  | 22 | 0.976 ^c^ |
|  |  |  | 125 | 0.976 ^c^ |
|  |  |  | 453 | 0.976 ^c^ |
|  |  |  | 807 | 0.976 ^c^ |
|  |  |  | 360 | 0.974 ^c^ |
|  |  |  | 608 | 0.974 ^c^ |
|  |  |  | 689 | 0.974 ^c^ |
|  |  |  | 726 | 0.974 ^c^ |
|  |  |  | 643 | 0.973 ^c^ |
|  |  |  | 818 | 0.973 ^c^ |
|  |  |  | 218 | 0.972 ^c^ |
|  |  |  | 238 | 0.972 ^c^ |
|  |  |  | 836 | 0.972 ^c^ |
|  |  |  | 772 | 0.971 ^c^ |
|  |  |  | 46 | 0.971 ^c^ |
|  |  |  | 60 | 0.971 ^c^ |
|  |  |  | 513 | 0.969 ^c^ |
|  |  |  | 355 | 0.969 ^c^ |
|  |  |  | 548 | 0.967 ^c^ |
|  |  |  | 102 | 0.967 ^c^ |
|  |  |  | 433 | 0.967 ^c^ |
|  |  |  | 735 | 0.967 ^c^ |
|  |  |  | 518 | 0.966 ^c^ |
|  |  |  | 414 | 0.965 ^c^ |
|  |  |  | 526 | 0.965 ^c^ |
|  |  |  | 456 | 0.96 ^c^ |
|  |  |  | 116 | 0.953 ^c^ |
|  |  |  | 448 | 0.952 ^c^ |
|  |  |  | 14 | 0.951 ^c^ |
|  |  |  | 322 | 0.951 ^c^ |
|  |  |  | 352 | 0.951 ^c^ |
|  |  |  | 399 | 0.951 ^c^ |
|  |  |  | 674 | 0.951 ^c^ |
|  |  |  | 708 | 0.951 ^c^ |
|  |  |  | 716 | 0.951 ^c^ |
|  |  |  | 749 | 0.951 ^c^ |
|  |  |  | 875 | 0.951 ^c^ |
| Chiapas | SLAC | 2 | 712 | 0.0370 ^b^ |
|  |  |  | 508 | 0.0963 ^b^ |
|  | FEL | 2 | 712 | 0.001 ^b^ |
|  |  |  | 508 | 0.043 ^b^ |
|  | FUBAR | 2 | 712 | 0.993 ^c^ |
|  |  |  | 508 | 0.982 ^c^ |

^a^ SLAC, single-likelihood ancestor counting; FEL, fixed effects likelihood; FUBAR, fast unconstrained bayesian approximation. ^b^ *p* – value. ^c^ posterior probability. ^d^ codon position is according to the fragment size.
