# Supplementary figures and images for: Genetic Variability of Chikungunya Virus in Southern Mexico
Source: Viruses. 2019 Aug 5;11(8):714. doi: 10.3390/v11080714 (PMC6722872; doi:10.3390/v11080714)

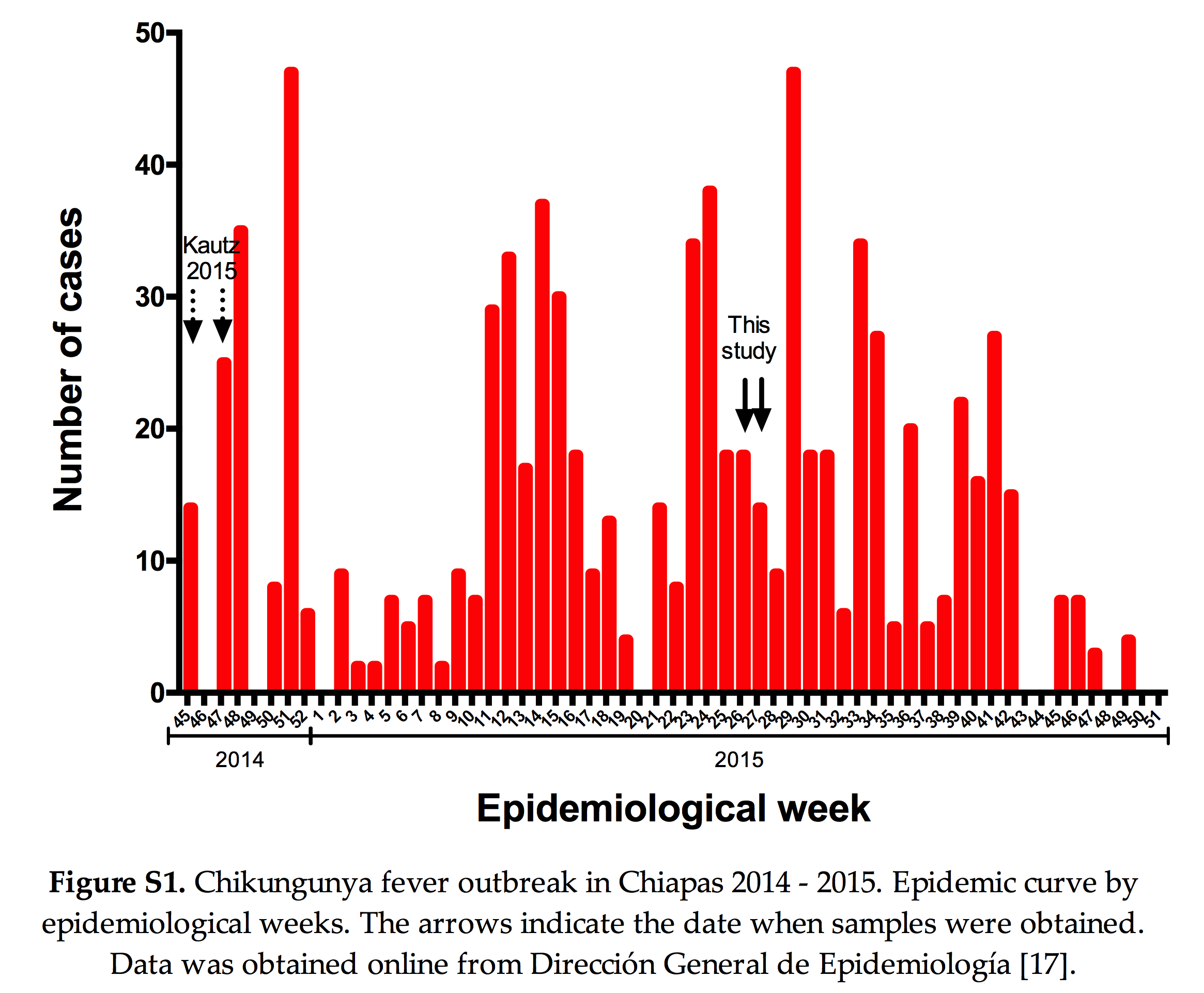

Supplement: Supplementary file 1 [file viruses-11-00714-s001.zip › Suplementary/FigureS1.tiff]

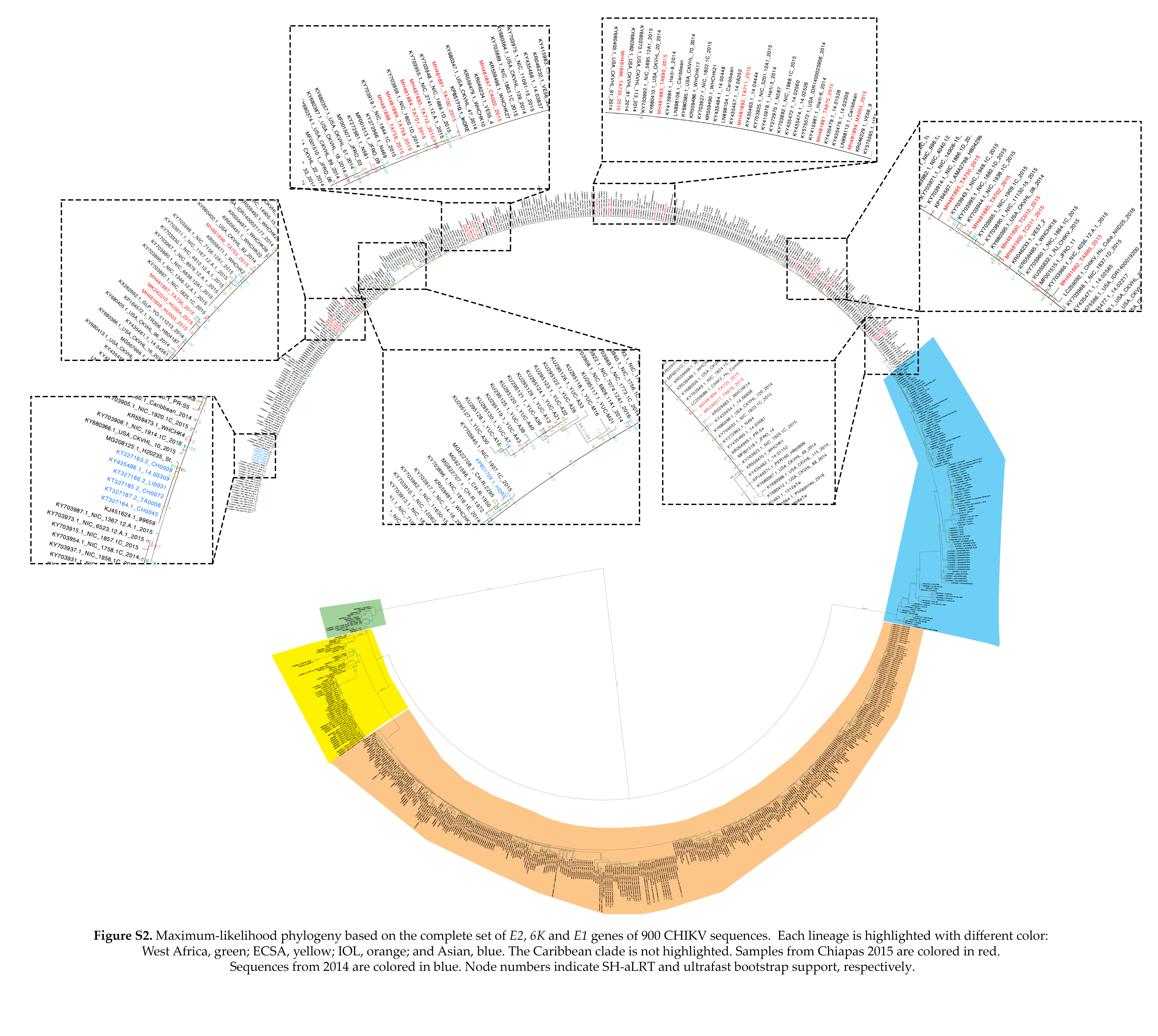

Supplement: Supplementary file 1 [file viruses-11-00714-s001.zip › Suplementary/FigureS2.tif]
